# Common metabolic networks contribute to carbon sink strength of sorghum internodes: implications for bioenergy improvement

**DOI:** 10.1186/s13068-019-1612-7

**Published:** 2019-11-20

**Authors:** Yin Li, Min Tu, Yaping Feng, Wenqing Wang, Joachim Messing

**Affiliations:** 1 Waksman Institute of Microbiology, Rutgers, The State University of New Jersey, Piscataway, NJ 08854 USA; 2 School of Agriculture and Biology, Shanghai Jiaotong University, 800 Dong Chuan Road, Shanghai, 200240 China

**Keywords:** Bioenergy, RNA-seq, Gene expression, Transcriptome analysis, Carbon partitioning, Sugar accumulation, Internode, Sorghum

## Abstract

**Background:**

*Sorghum bicolor* (L.) is an important bioenergy source. The stems of sweet sorghum function as carbon sinks and accumulate large amounts of sugars and lignocellulosic biomass and considerable amounts of starch, therefore providing a model of carbon allocation and accumulation for other bioenergy crops. While omics data sets for sugar accumulation have been reported in different genotypes, the common features of primary metabolism in sweet genotypes remain unclear. To obtain a cohesive and comparative picture of carbohydrate metabolism between sorghum genotypes, we compared the phenotypes and transcriptome dynamics of sugar-accumulating internodes among three different sweet genotypes (Della, Rio, and SIL-05) and two non-sweet genotypes (BTx406 and R9188).

**Results:**

Field experiments showed that Della and Rio had similar dynamics and internode patterns of sugar concentration, albeit distinct other phenotypes. Interestingly, cellulose synthases for primary cell wall and key genes in starch synthesis and degradation were coordinately upregulated in sweet genotypes. Sweet sorghums maintained active monolignol biosynthesis compared to the non-sweet genotypes. Comparative RNA-seq results support the role of candidate *Tonoplast Sugar Transporter* gene (*TST*), but not the *Sugars Will Eventually be Exported Transporter* genes (*SWEETs*) in the different sugar accumulations between sweet and non-sweet genotypes.

**Conclusions:**

Comparisons of the expression dynamics of carbon metabolic genes across the RNA-seq data sets identify several candidate genes with contrasting expression patterns between sweet and non-sweet sorghum lines, including genes required for cellulose and monolignol synthesis (*CesA*, *PTAL,* and *CCR*), starch metabolism (*AGPase*, *SS*, *SBE,* and G6P-translocator *SbGPT2*), and sucrose metabolism and transport (*TPP* and *TST2*). The common transcriptome features of primary metabolism identified here suggest the metabolic networks contributing to carbon sink strength in sorghum internodes, prioritize the candidate genes for manipulating carbon allocation with bioenergy purposes, and provide a comparative and cohesive picture of the complexity of carbon sink strength in sorghum stem.

## Introduction

A characteristic feature of vascular plants is that CO_2_ is fixed by photosynthesis in source leaves and then transported to and utilized by different sink organs for growth. During this process, three key factors can affect source-to-sink relationship: (i) photosynthesis capacity that determines carbon availability; (ii) sugar transportation; (iii) carbon utilization and storage at sink organs [1]. During plant growth and development, sink organs/tissues are dynamic [2, 3]. For example, immature leaves and shoot apical meristems are sink organs in vegetative stages while developing flowers and seeds become sinks in reproductive stages. Therefore, the abilities of sink organs to obtain, utilize, and store carbon (so-called ‘sink strength’) are dynamic and tightly controlled [4, 5]. Moreover, the distribution of carbon utilization/storage (carbon allocation) within a sink tissue is well coordinated. The C4 grasses include important bioenergy crops, such as maize, sorghum, switchgrass, and sugarcane, and serve as the most-significant plant source of carbohydrates and bioethanol [6]. Among these C4 crops, *Sorghum bicolor* is an excellent example for studying carbon allocation, because sweet sorghum varieties have two sink organs, seeds, and stem [7, 8]. While a significant portion of carbon reserves are in cell wall components, large amounts of soluble sugars (primarily sucrose) and starch accumulate in sorghum stems after flowering. Thus, this feature makes sweet sorghum an interesting model to study carbon partitioning and sugar accumulation for other bioenergy crops like sugarcane [9, 10]. In addition, sweet sorghum can accumulate considerable amount of starch in the internode [11] and has differential expression patterns of the cell wall-related genes compared to non-sweet genotypes [12], indicating that the distribution of carbon utilization within sweet sorghum internodes may be redirected to establish sink strength. Also, sorghum is an emerging bioenergy crop with multiple advantages: (i) a ~ 730-Mb diploid genome and several reference assemblies with great synteny to maize and sugarcane [13–16]; (ii) good tolerance to several abiotic stresses and desirable agronomical features, such as the stay-green trait [7, 17, 18]; (iii) rich genetic resources [19], such as several EMS resources [20–22]; (iv) ability to be transformed and genome-edited [23, 24]; (iv) potential in phytoremediation of soil pollution [25].

Knowledge of sorghum stem sugar accumulation has accrued from genetics, physiology, molecular biology, and omics studies. Sucrose starts to increase after internode elongation, with a dramatic accumulation from anthesis to the first 2 weeks post-anthesis [12, 26]. Population genetics studies revealed that stem sugar yield is determined by three factors: stem juiciness, stem biomass-related traits, and sugar concentrations of the juice, the first two affecting juice volume/weight [27, 28]. Sorghum stem juiciness is largely controlled by a single gene, named *Dry* culms (*D*) [29], which encodes an NAC transcription factor controlling programmed cell death of stem parenchyma cells, thereby affecting secondary cell wall compositions [30–33]. While quantitative trait loci (QTL) associated with sugar-related traits have been reported in sorghum [34–39], the molecular mechanism regulating stem sugar concentrations remains unclear. Physiology results using radiolabeling and dye transport approaches suggest that sucrose may be transported to storage parenchyma via apoplasmic and/or symplasmic routes [40–43].

Carbohydrates are stored in sorghum stems in three significant forms, sucrose in vacuoles, starch in plastids, and lignocellulosic cell wall biomass [26]. The sucrose in vacuoles could be related to several sugar transporters, such as *Sucrose Transporters* (*SUT*s), *Tonoplast Sugar Transporters* (*TST*s), and *Sugars Will Eventually be Exported Transporters* (SWEETs). The expression profiles of these transporters have been examined in sweet and grain genotypes [12, 42–46], suggesting *SbTST2* as a candidate gene for stem sugar difference between sweet and grain sorghum lines [46]. The sorghum SWEETs fell into the four phylogenetically defined clades, in which evidence of phylogeny–function correlation has been shown in several species [47–54]. Starch synthesis requires a suite of well-characterized enzymes and transporters (reviewed previously in [55, 56]), including ADP-glucose pyrophosphorylase (AGPase), soluble starch synthase (SS), granule-bound starch synthase (GBSS), starch branching enzyme (SBE), starch debranching enzyme (DBE)/isoamylase (ISA), and glucose-6-phosphate translocators (GPT) that fuel starch synthesis with glucose-1-phosphate (G1P) [57]. Starch is degraded by a set of kinases and hydrolases, including glucan–water dikinase (GWD), phosphor-glucan–water dikinase (PWD), α- and β-amylase (AMY and BAM, respectively) and disproportionating enzyme (DPE) [58–60].

Plant cell walls include primary and secondary cell wall (PCW and SCW, respectively). PCW, mainly composed of cellulose, hemicellulose, and pectin, exists in all plant cell types and is tensile to yield to cell expansion and turgor pressure. SCW, mainly composed of lignin, crosslinked with cellulose and hemicellulose, exists in specific cell types to provide mechanical support and serve as a defensive barrier. Cellulose, as the most abundant structural polysaccharide in plant cell wall, is synthesized by cellulose synthases that are encoded by *CesA* gene family [61, 62], of which two phylogenetic groups are responsible for PCW and SCW biosynthesis, respectively [63]. Hemicelluloses are branched hetero-carbohydrate polymers synthesized by cellulose synthase-like (Csl) enzymes. Lignin is a complex heteropolymer crosslinked from three monolignins, namely *p*-coumaryl (H), coniferyl (G), and sinapyl (S) alcohols [64]. Ten major gene families required for monolignol biosynthesis have been well studied in sorghum at the genome level [65], namely, phenylalanine ammonia-lyase (PAL), cinnamate 4-hydroxylase (C4H), 4-coumarate:CoA ligase (4CL), hydroxycinnamoyl-transferase (HCT), 4-coumarate 3-hydroxylase (C3H), cinnamyl-CoA reductase (CCR), cinnamyl alcohol dehydrogenase (CAD), caffeic acid *O*-methyltransferase (COMT), caffeoyl-coenzyme A 3-*O*-methyltransferase (CCoAOMT), and ferulate 5-hydroxylase (F5H). Seven out of the ten enzymes have been structurally and biochemically investigated (see “Methods”). Three *Brown midrib* (*Bmr*) loci are known to encode enzymes of monolignol biosynthesis [66].

Recent studies of sorghum stem sugar accumulation have expanded to gene and genome levels, including comparisons between grain and sweet sorghum using whole-genome re-sequencing [67, 68] and mRNA and small RNA transcriptome analyses [69–72]. RNA-seq data of sugar-accumulating internodes have been reported in three sweet sorghum genotypes [12, 26, 45]. McKinley et al. focused on the expression of biosynthetic genes of cell wall components across reproductive development in sweet sorghum Della [26] and expanded their analysis to starch metabolism, demonstrating the upregulation of starch-metabolic genes and the tendency of higher starch contents in sweet genotypes [11]. Mizuno et al. [45] focused on SWEET transporters in Japanese sweet sorghum SIL-05. Li et al. integrated a time-series of transcriptome and metabolome data of sorghum stems [12] and revealed that: (1) carbon sink strength in stem is related to the coordination of several primary metabolic pathways, and (2) the sucrose signal, trehalose-6-phosphate (T6P), may be involved in stem sugar accumulation. However, important biological questions remain unanswered: (1) Are the afore-mentioned candidate pathways/genes specific for a given sweet genotype? (2) What are the common expression features of primary metabolism and sugar transport among different sweet sorghum lines? Several previous studies either focused on a particular aspect of carbohydrate metabolism or a gene family [11, 26, 45]. A single omics data set is insufficient to answer the above questions and is unable to provide a comprehensive and cohesive molecular view of carbon partitioning and sink strength in sorghum stem. To address these issues, we performed a detailed comparative transcriptome analysis among the three RNA-seq data sets with a focus on sucrose-related metabolic pathways and also characterized the sugar accumulation dynamics of the sweet sorghums Rio and Della. Here, we report that the three sweet genotypes have similar expression profiles in key genes involved in carbon utilization pathways and sucrose transport, highlighting the reliable candidate genes from the *TST* family, *CesA* family, and starch-metabolic and monolignol biosynthetic pathways with common expression profiles among sweet genotypes. We question the involvement of the potential SWEET candidates based on their non-consensus expression patterns and phylogeny–function correlation.

Our analysis allows us to propose a common metabolic network for carbon partitioning and sink establishment in sweet sorghum internodes. The common network implies that: (1) the major carbon reserves reflected by the analyzed primary metabolic pathways and sucrose transportation jointly contribute to the sink strength of stem tissue; (2) the activities of the primary metabolic pathways reflect the distribution of carbon utilization in the stem; (3) carbon allocation could be changed by manipulating the identified candidate genes and, hence, the corresponding carbon reserves to improve stem carbohydrate compositions for bioenergy purposes.

## Methods

### Plant materials and field experiments

Three sweet sorghum genotypes, Rio, Della, and SIL-05, were used (Additional file 1). Rio (PI 651496) is developed from a cross of Rex (PI 641835) and Manawan (PI 152959) [73], whereas Della is developed from a cross of Dale (PI 651495) and ATx622 [74]. SIL-05 is a Japanese sweet sorghum line developed from a cross of BR504 and Brown Native by Kyushu, Okinawa Agricultural Research Center of NARO (National Agricultural and Food Research Organization. http://www.naro.affrc.go.jp/patent/breed/0500/0509/001471.html).

Rio and Della were obtained from the United States Department of Agriculture National Plant Germplasm System (USDA-NPGS) and phenotyped at the Waksman experimental field (Piscataway, NJ) in 2018. SIL-05 was not phenotyped due to unavailable seeds from USDA-NPGS. Rio and Della were grown in each 8-row plot, with each row containing ten germinated plants. Only the six central plants per row from the six central rows were used for phenotyping to minimize border effect. Plant height, number of above-ground internode, days to flower, and internode total sugar concentration were recorded at six stages, including anthesis, 10, 18, 22, 32, and 38 days after flowering (DAF) to capture the sugar concentration dynamics. Internode fresh weight and dry weight were measured at five stages (from 10 to 38 DAF). Dry weight was measured after internode samples were dried at 65 °C for 96 h. As an indicator of juice volume, internode water content was calculated as previously described [33]. The total sugar concentration of internode-extracted juice was measured by Brix [75]. All internode samples were collected in the field at 9:00–11:00 AM and stored on ice. After transferring samples back to the laboratory, the juice was extracted immediately.

### RNA-seq data analysis

Three RNA-seq data sets were used (Additional file 2). The first data set is the transcriptomes of sugar-accumulating internodes from a conversion line R9188 and its two parents Rio and BTx406 collected at flag leaf stage, flowering and 10 and 15 days after flowering (designated as T1, T2, T3 and T4, respectively) [12]. The dwarf inbred line R9188 was developed from the BTx406/Rio cross followed by one backcross to Rio and contains the early flowering and dwarf loci introgressed from BTx406 [76]. RNA was extracted from the pooled tissues from upper internodes of Rio, BTx406 and R9188 (internode 2, 3, and 4, numbered from top to bottom) as described elsewhere [12].

The second RNA-seq data set is the transcriptomes of Della internodes collected from eight developmental stages (29, 16, and 7 days before anthesis, anthesis, and 11, 25, 43, and 68 days after anthesis, designated as A-29, A-16, A-7, A0, A11, A25, A43, and A68, respectively) [26]. Particularly, Della stem was fully mature at A-7 and the grains reached a soft dough stage at A25 and became completely mature before A43. RNA was extracted using the tenth internode of greenhouse-grown plants (numbered from bottom to top), while field grown Della in Texas, US had 14–15 internodes as previously described [26].

The third RNA-seq data set is the SIL-05 transcriptomes of internode, panicle, and leaf tissues at three stages, 1, 17, and 36 days after heading (1 DAH, 17 DAH, and 36 DAH, respectively) [45]. SIL-05 flowers between 1 and 17 DAH, and sucrose starts to accumulate in SIL-05 stem between 1 and 36 DAH and can reach 18.9% in juice at 64 DAH. RNA was extracted from the corresponding internode from the leaf below flag leaf of SIL05 as described previously [45, 77].

**Fig. 1:**
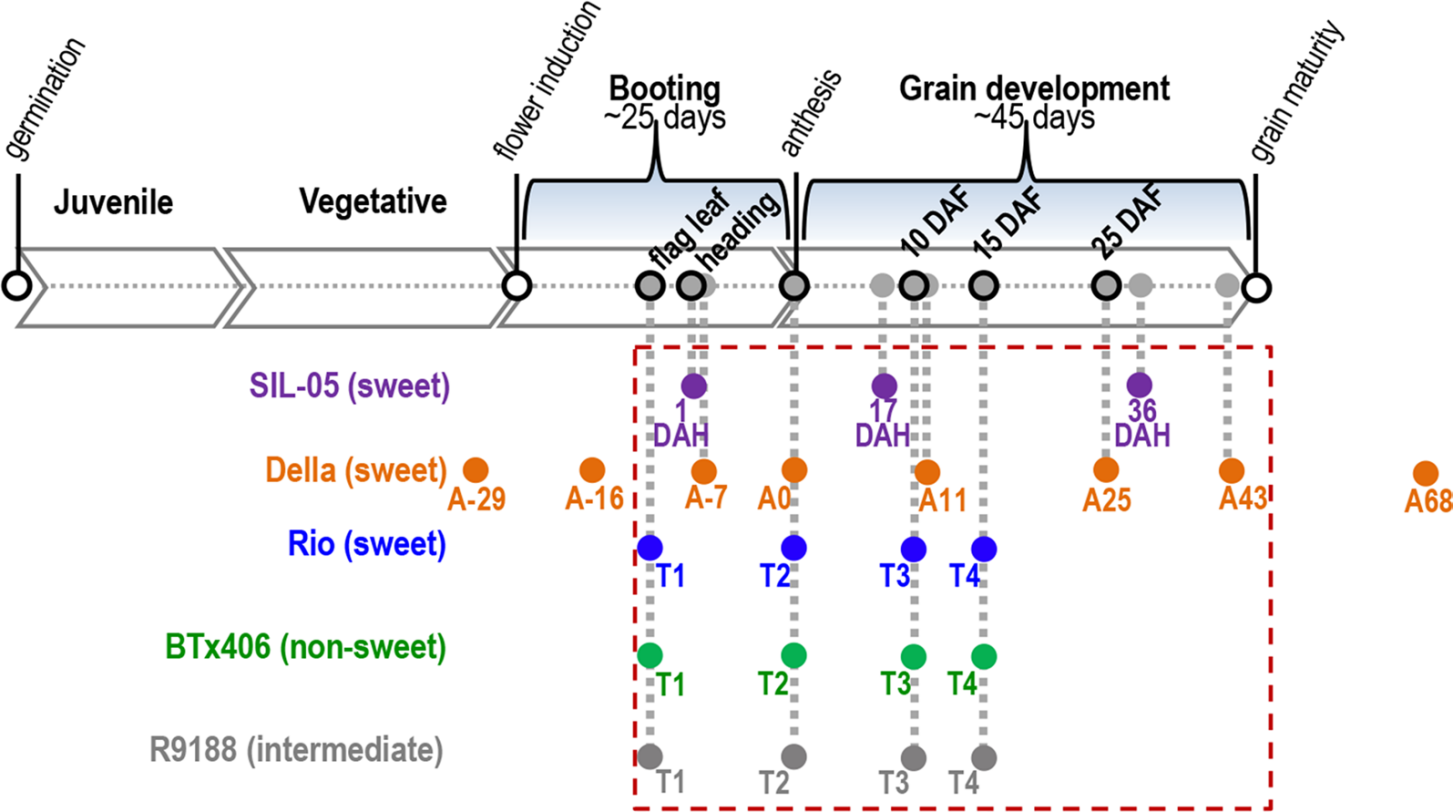
Time-course alignment of the RNA-seq time points between sorghum genotypes. Duration of booting and grain development stages were according to the field observations for Rio and Della, and previous studies [12, 26, 45, 77]. The time points when the RNA-seq samples were collected are color-coded by genotypes and aligned to the developmental process of sorghum, with those samples related to stem sugar accumulation highlighted in the red broken box

The stages of the three RNA-seq data sets were aligned relative to anthesis and the stages over stem sugar accumulation were identified for each genotype (Fig. 1). To overcome the issues of lacking replicates (for dataset3) and unavailability of raw data (for dataset2), we took the following strategies for analysis (Additional files 3, 4). (1) The raw data of data sets 1 and 3 were quality filtered and analyzed using the same pipeline. Reads were mapped to the sorghum reference genome (BTx623, Sbicolor_v2.1_255) using TopHat v2.0.14 (a maximum mismatch of 9 bp and default settings for other parameters) [13, 78]. Read counts were calculated using ‘HTseq’ with uniquely mapped reads and RPKM values were calculated for SIL-05 [79]. (2) The dataset2 expression matrix using reads per kilobase of transcript per Million mapped reads (RPKM) were reversely calculated to the normalized average read counts for three replicates of each time point based on RPKM definition with the assumption that in Della, every gene has the same gene length as that in the genotypes from data sets 1 and 3. Then, the normalized average read counts can be considered as an input of read count matrix without replicates for differential expression analysis. (3) To perform differential expression analysis for data sets 2 and 3 without replicates, the gene-wise dispersion of biological variation (using biological coefficient of variance, BCV, as index) for sorghum internode tissues was calculated using the triplicates of dataset1 with “edgeR” [80]. The BCV matrix was used to identify differentially expressed genes in data sets 2 and 3 with “edgeR” using the following criteria: *q* values < 0.05 and log_2_Fold Change (log_2_FC) ≥ 1. (4) To investigate the sample relationship across data sets, potential batch effects for the expression matrix between the three RNA-seq studies were minimized with quantile normalization using ‘preprocessCore’ as described in Additional file 4 [81, 82].

To identify common candidate genes associated with stem sugar accumulation in all the data sets, we investigated genes involved in primary metabolism and sugar transporters, and considered candidates using the following criteria. (1) Low-expression genes were excluded (maximum RPKM ≤ 5), because the genes are annotated as encoding enzymes or transporters functioning in primary metabolic pathways that are responsible for major carbon reserves in sorghum stem. (2) To identify genes showing distinct expression trends between sweet and non-sweet genotypes, the genes should meet two criteria: (2A) differential expression at post-anthesis stages compared to anthesis or pre-anthesis stages in the sweet genotypes, but not in the non-sweet genotypes, or vice versa; (2B) an expression trend in Della and SIL05 similar to that in Rio, but contrasting to BTx406/R9188. To visualize the similar expression trends of selected candidate genes in sweet versus non-sweet comparison, heatmap of log_2_ fold change was used with the fold changes calculated using RPKM + 0.1 to avoid zero values.

### Identification of genes involved in primary metabolism and sugar transport

Annotation information of genes potentially involved in cell wall metabolism, starch and sucrose metabolism, and glycolysis and sucrose transporter families (SUTs, SWEETs, and TSTs) was extracted from earlier literature and databases [83–89]. Detailed methods for gene annotation are in Additional file 4. All the gene annotation information is shown in Additional file 5.

### Phylogenetic analysis of SWEET gene family

The 23 SWEETs reported previously were used for gene family analysis (Additional file 6). To re-confirm that these genes encode putative SWEETs, BLAST searches against sorghum genomes v2 and v3 followed by filtering using two MtN3 domain (Pfam: PF03083) were performed [15, 47]. The deduced amino acid sequences of sorghum SWEETs were compared with rice and maize SWEET proteins; only primary or canonical transcripts were used. The rice and maize SWEETs were described previously [52–54]. *SbSWEET* nomenclature was according to Bhimidine et al. [46]. Sequence alignment was performed using MUSCLE and neighbor joining (NJ) phylogenetic trees were generated using MEGA v7 with JTT protein substitution model, pairwise deletion for gaps/missing data, and 1000-time bootstrap [90]. Sobic.003G038800 was not included in phylogenetic tree due to its two incomplete MtN3 domain (Additional file 7). Sorghum expression atlas and MOROKOSHI database were used to evaluate the spatio-temporal expression patterns of SWEETs [15, 84].

### Quantitative PCR validation

Total RNA was extracted from the pooled samples of upper internodes (internodes 2, 3, and 4, numbered from top to bottom) for Rio, BTx406, and R9188, respectively, using TRIZOL and PureLink RNA extraction kit (Invitrogen). The samples were collected from the plants grown in a split-plot design and are the same samples used for RNA-seq of Rio/R9188/BTx406 as described previously [12]. The concentration and purity of the RNA were evaluated using a Nanodrop 2000 spectrophotometer. After cDNA synthesis with SuperScript III First Strand kit, real-time quantitative PCR (qPCR) was conducted with PowerUp SYBR Green mastermix (Thermo Fischer) using the ABI StepOne Plus Real-Time PCR system. Relative expression levels were calculated using the ΔΔCT method with Ubiquitin as the internal reference gene because of its stable expression determined by the RNA-seq data [12]. All real-time qPCR primers are listed in Additional file 8.

## Results

### Dynamics of internode sugar accumulation

**Fig. 2:**
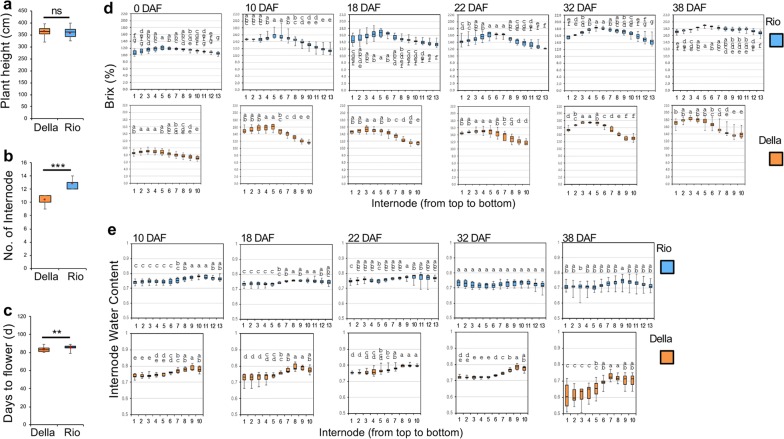
Comparison of the phenotypes and dynamics of sugar accumulation between Della and Rio. Three phenotypes, including plant height (**a**), number of above-ground internode (**b**), and days to flowering (**c**), were compared between Della and Rio. The statistical differences determined by Welch two-sample *t* test and presented using asterisks (*, **, *** indicate *p *< 0.05, *p *< 0.01 and *p *< 0.005, respectively). **d** Dynamics of total sugar concentration (measured by Brix) from 0 DAF (anthesis) to 38 DAF between Della and Rio. **e** Dynamics of internode water content from 10 DAF to 38 DAF between Della and Rio. The statistical differences in Brix (**d**) and internode water content (**e**) between internodes for each genotype and time point were calculated using one-way ANOVA and multiple comparison, and displayed by letter. The values within a genotype and time point labeled by the same letter are not significantly different at *p *= 0.05. For **a**–**c**, *n* = 36; for **d**, **e**, *n* = 6

We compared the dynamics of internode sugar accumulation between Della and Rio, which had similar plant heights but differed in above-ground internode number (Fig. 2a, b). Both genotypes showed slight difference in days to flowering (Fig. 2c). Della and Rio showed similarities in internode sugar concentration dynamics in several aspects. First, the total sugar concentrations were markedly increased in both genotypes from anthesis to 38 DAF (from ~ 9 to ~ 18% in Della and from ~ 12 to ~ 19% in Rio; Fig. 2d). Second, the upper internodes (internode 2–8 for Rio and internode 1–5 for Della) had higher sugar concentrations than the lower internodes in both genotypes. Third, total sugar concentrations were significantly increased at two stages in both genotypes: (i) the first 10 days after anthesis and (ii) from 22 to 32 DAF (Additional file 9). We also characterized the water content dynamics of both genotypes from 10 to 38 DAF (Fig. 2e; Additional file 9). Della and Rio had juicy stems and their internode water contents remained stably high (~ 70% to 80%) during stem sugar accumulation, except for an obvious decrease at 38 DAF in Della. Slight, but statistically significant, decreases in water contents were observed at 32 DAF for most of the internodes in both genotypes, matching slightly increased Brix levels. Generally, the upper internodes had lower water contents compared to lower internodes (Fig. 2e).

### Comparative transcriptome analysis of sugar-accumulating stems

**Fig. 3:**
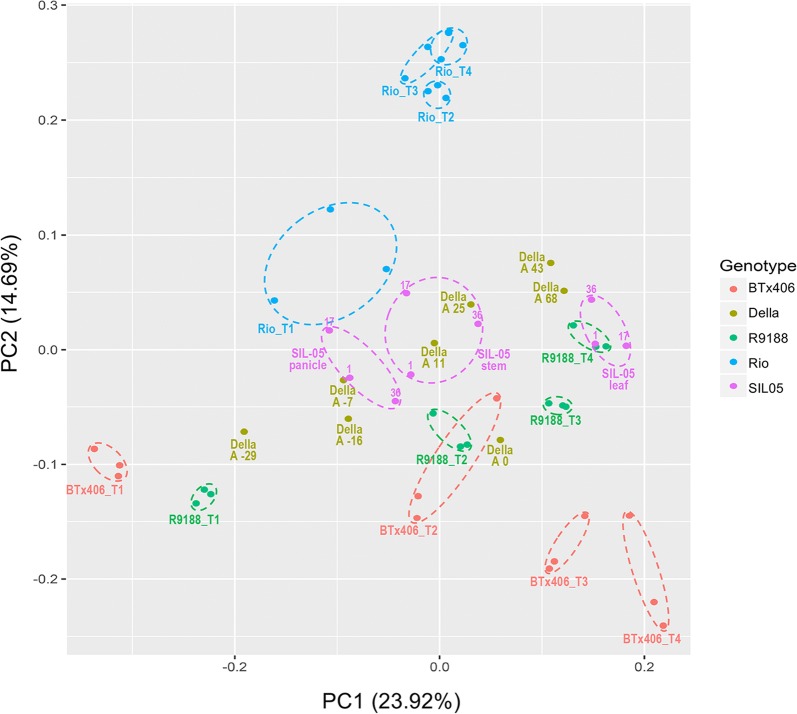
PCA analysis. PC1 and PC2 variance of expression between RNA-seq samples and related developmental stages and genotypes, respectively, is shown

Batch effects between the three RNA-seq data sets were removed (see “Methods”, Additional files 4 and 10). The developmental timelines for the five genotypes were aligned relative to their anthesis (Fig. 1) [12, 26, 45, 77]. Principal component analysis (PCA) showed that ~ 40% of transcriptome variances between samples were explained by PC 1 and 2, which appeared to be associated with developmental stages and genotypes, respectively (Fig. 3). Our previous study shows that the Rio-converted line R9188 is partially active in primary metabolism and has an intermediate stem sugar concentration. The PCA results showed that R9188 stem transcriptomes were differentiated from those of Rio and BTx406. Della stem transcriptomes fell between Rio and R9188, with SIL-05 stem transcriptomes grouped close to Della A11 and A25. Similarly, hierarchical clustering (HC) results (Additional file 11) grouped the transcriptomes into five clusters and identified those enriched with sugar-accumulating internodes: (i) some developmental stages of Della, when soluble sugars are actively accumulated, were clustered with Rio stems (cluster 3); (ii) SIL-05 stem samples were clustered with R9188 stems (cluster 4). Overall, PCA and HC results suggest that metabolic active stem samples with high or intermediate sugars tend to group together.

### Cellulose synthetic genes

Non-structural carbohydrates (sugars/starch) and structural carbohydrates (cell wall components) represent major carbon reserves in the stem during post-anthesis. Sugars and starch together account for ~ 50% of stem dry weight, whereas structural carbohydrates account for ~ 30% [26]. Representative sweet varieties had higher starch content (ranging from ~ 3 to 10%) than grain sorghum lines (< 2%), supporting starch as an important carbon reserves in stem [11]. We compared the expression dynamics of primary metabolic genes to examine whether a similar expression trend could be observed in Della and SIL05 for the gene that was differentially expressed between Rio and BTx406/R9188 (Additional files 5 and 12). While considering the quantitative expression differences between genotypes, we primarily focused on the fold changes of gene expression within a genotype due to limitations in quantitative cross-comparison between data sets.

**Fig. 4:**
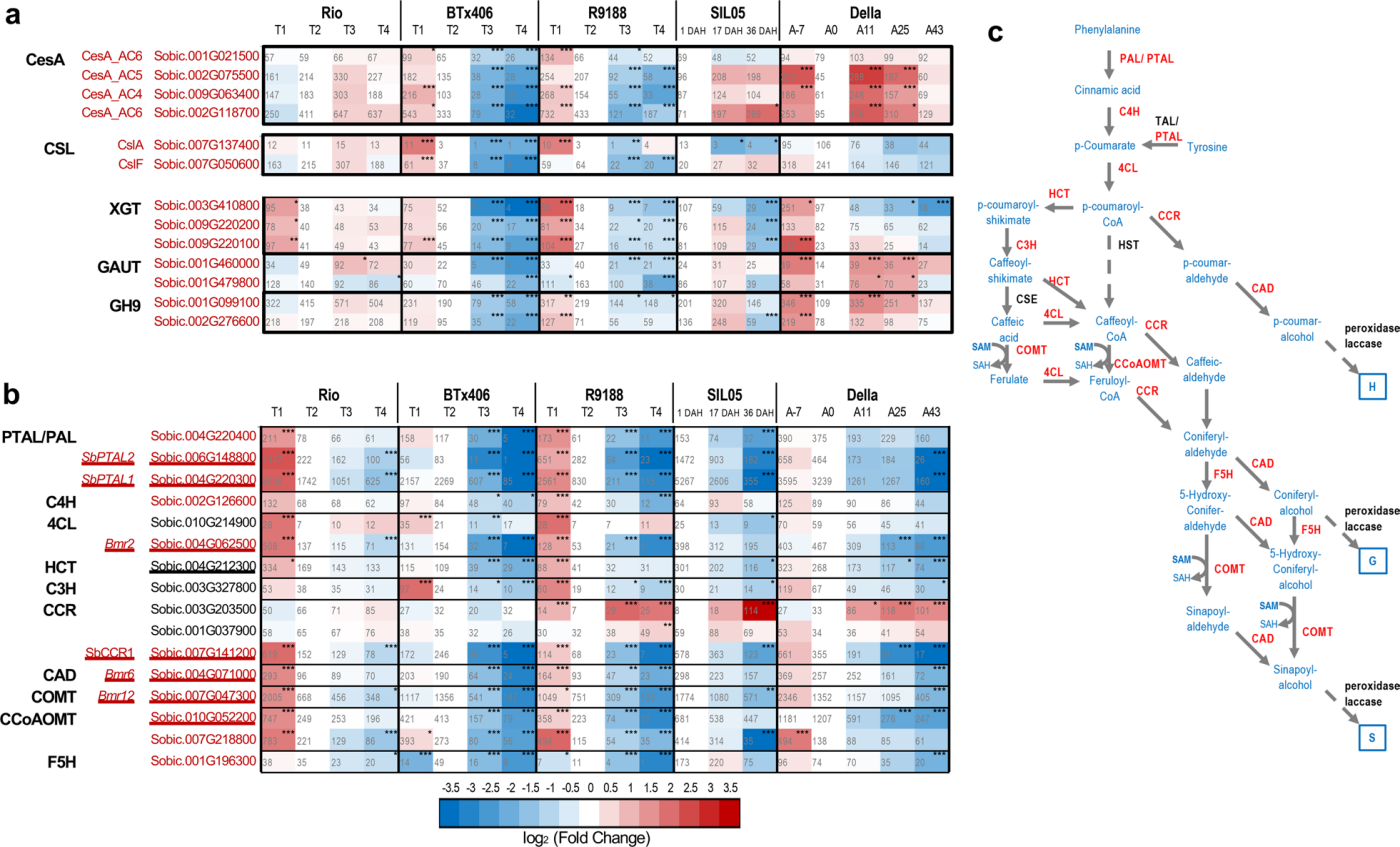
Comparison of representative genes involved in cell wall metabolism between sorghum genotypes. Gene expression dynamics are shown in heatmaps for cell wall biosynthetic genes (**a**) and monolignol biosynthetic genes (**b**), with the pathway of monolignol biosynthesis shown in **c**. The cell colors are shaded to reflect the magnitude of log_2_ fold change of gene expression relative to the anthesis stages in each genotype. The expression levels in RPKM are labeled on each cell with statistical differences (*q* values determined by edgeR) indicated by asterisk (*q < 0.05; **q < 0.01; ***q < 0.005). The geneIDs highlighted in red are those that share the similar expression trends between sweet lines Rio, Della and SIL05 but contrast to BTx406 and R9188. In **b**, the monolignol biosynthetic genes with functional evidence in sorghum are underlined, with their gene names or corresponding sorghum mutants labeled according to previous studies [99–107]

Several *CesA* genes were highly expressed in sweet sorghum genotypes, but significantly decreased in BTx406/R9188 after flowering (Fig. 4a). These *CesA* genes belong to the ancestral clusters CesA_AC4, CesA_AC5, and CesA_AC6 in phylogeny and are associated with PCW cellulose synthesis [63]. In contrast, the expression of *CesA* genes corresponding to SCW cellulose synthesis (Sobic.001G224300 and Sobic.002G205500) decreased post-anthesis in all genotypes. Several *Csl* genes were differentially expressed between sweet and non-sweet genotypes. A *CslF* gene that has a major role in synthesis of mixed-linkage (1,3; 1,4) β-glucan (MLG) maintained its expression levels in the sweet genotypes with particularly high expression in Della and Rio, but decreased from pre-anthesis stages in non-sweet genotypes [73, 91]. Similarly, a CslA gene (Sobic.007G137400) decreased its expression in BTx406/R9188, but maintained a seemingly higher expression levels in sweet genotypes [92]. Its homologs in *Arabidopsis* are both responsible for mannan synthesis and affect cell wall integrity and organization [93]. Two highly expressed genes that encode homogalacturonan α-1,4-galacturonosyltransferase (GAUT) and are possibly responsible for pectin synthesis were significantly decreased in BTx406/R9188 after flowering, but were either stable or upregulated compared to anthesis stage in sweet sorghum [92]. Three xyloglucan galactosyltransferase (XGT) genes exhibited similar trends: their expression levels in BTx406/R9188 were significantly downregulated when comparing to the anthesis stage, but such downregulation was not observed after flowering in the sweet genotypes. More interestingly, two highly expressed genes encoding endo-1,4-β-glucanase (CAZy ID: GH9) were decreased in BTx406/R9188, but remained high or upregulated expression in sweet genotypes after flowering. The *Arabidopsis* homolog (AT5G49720, *KOR1*) of the two GH9 genes can interact with CesA complex and is required for cellulose deposition [94–96], while the other *Arabidopsis* homolog (AT1G19940) affects cell wall crystallinity, secondary cell wall development, and biomass of Arabidopsis plants [96]. Overall, the genes highlighted here, *CesA*, *Csl*, *XGT*, *GAUT,* and *GH9*, are important for the synthesis of cellulose, pectin, and hemicellulose (including xyloglucation, mannan, and MLG), which are the major components of primary cell wall [92]. Also, MLG is suggested as a storage form of stem glucose when comparing the carbon allocation differences between grain, sweet, and wild sorghum lines [97, 98]. The expression analysis of cellulosic genes suggests that sweet sorghum stem tissue maintains an active primary cell wall development during post-anthesis that could serve as a significant carbon demand.

### Monolignol biosynthetic genes

Extensive functional and structural studies in sorghum have identified and characterized the major genes controlling key steps of monolignol biosynthesis. These genes include those encoding the first and the third enzymes in the phenylpropanoid pathway (PAL and 4CL, respectively) that impact on the metabolic flux of monolignol precursors [99, 100], and several downstream genes encoding the enzymes (HCT, CCR, CAD, COMT, and CCoAMOT) that alter overall lignification and/or monolignin ratios [101–107]. Here, the RNA-seq data sets confirmed that these major functional genes are among those with the highest expression levels in their own families (Fig. 4b, c). The monolignol pathway was active in all the genotypes before flowering and was gradually downregulated after stem maturation. Sweet genotypes had higher expression levels of these genes during post-anthesis compared to BTx406/R9188, with statistical differences observed between Rio versus BTx406/R9188.

**Fig. 5:**
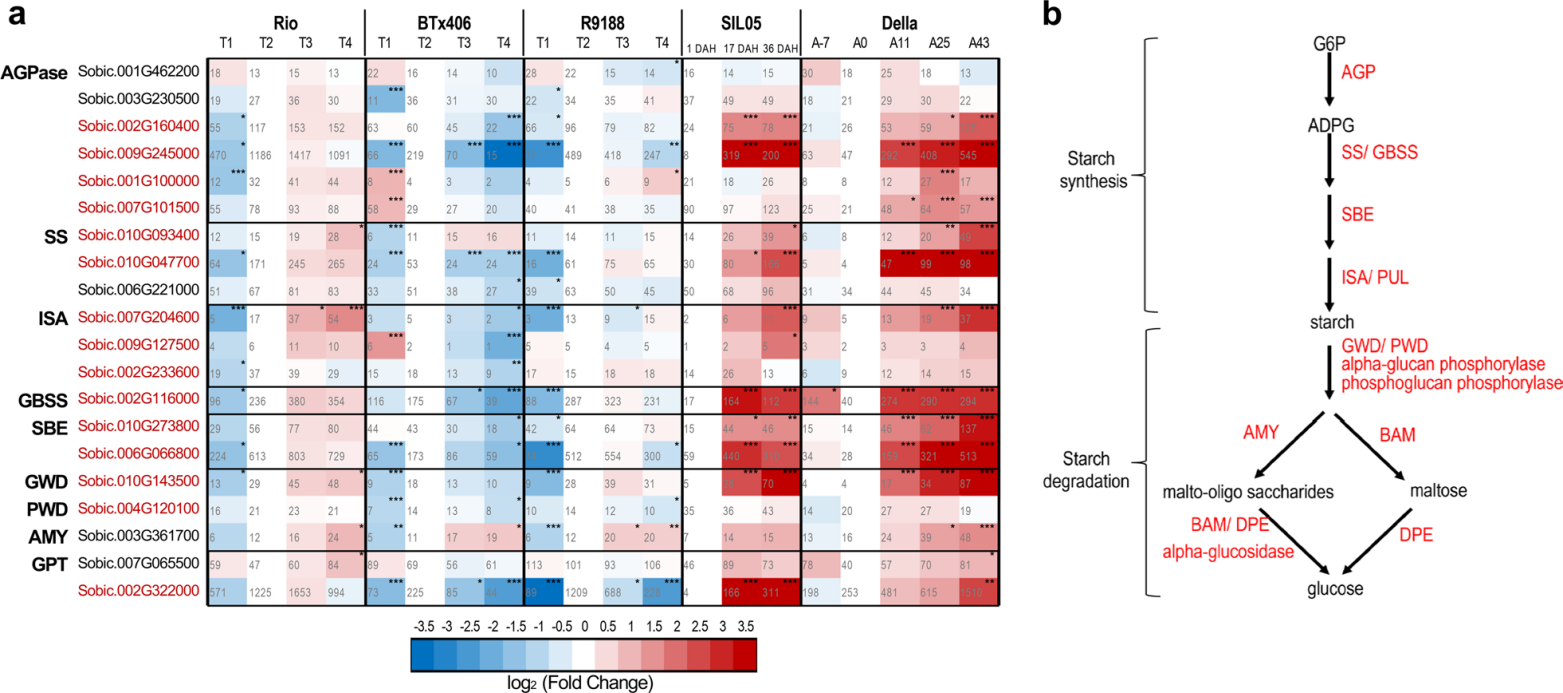
Comparison of starch-metabolic genes between sorghum genotypes. The expression dynamics of representative starch-metabolic genes were compared between sorghum genotypes and shown in heatmap (**a**), with the starch-metabolic pathway shown in **b**. The cell colors are shaded to reflect the magnitude of log_2_ fold change of gene expression relative to the anthesis stages in each genotype. The expression levels in RPKM are labeled on each cell with statistical differences (*q* values determined by edgeR) indicated by asterisk (*q < 0.05; **q < 0.01; ***q < 0.005). The geneIDs highlighted in red are those shared the upregulation expression trends between sweet lines Rio, Della and SIL05 but contrast to BTx406 and R9188

Previous studies on sorghum *PAL* genes have identified two subgroups encoding the enzymes active for l-Phe deamination (PAL) and l-Phe/-Tyr deamination (PTAL), respectively [100]. Expression profiling showed that two *SbPTAL*s (Sobic.004G220300 and Sobic.006G148800) and one *SbPAL* (Sobic.004G220400) had the highest expression levels in stem and their expression decreased remarkably during post-anthesis in BTx406/R9188. In contrast, the *SbPTAL*s and *SbPAL* maintained their expression levels from ~ 50 to several hundreds of RPKM in sweet genotypes, significantly higher than those in BTx406/R9188 (Fig. 5a). Consistent with the higher expression of *SbPTAL*s in sweet sorghum, l-tyrosine content was decreased in BTx406/R9188, but remained stable over time in Rio [12] (Additional files 4, 13), which could support the metabolic flux of phenylpropanoid biosynthesis in sweet sorghum. The other two enzymes that could influence the metabolic flux of precursors of flavonoids and lignins are C4H and 4CL [99, 100]. Indeed, the most highly expressed *C4H* gene (Sobic.002G126600) was significantly decreased in BTx406/R9188 compared to Rio at 15 DAF but was stable in the sweet genotypes. The predominant *4CL* gene (Sobic.004G062500, *Bmr2*) exhibited an expression pattern similar to *C4H*: its expression started to decrease in BTx406/R9188 at 10-days after flowering (T3), while in sweet genotypes, it remained stable until 15–25 days after flowering (for Rio and Della, respectively).

For the genes encoding downstream enzymes in the monolignol pathway, expression of several major functional genes including *CCR*, *CAD*, *COMT*, *CCoAOMT,* and *F5H* was decreased from 10 DAF in BTx406/R9188, but such an expression trend was not detected until 15–25 DAF in sweet genotypes (Fig. 4b). Several of these predominantly expressed genes encode the functional enzymes for each family, of which the structural information and substrate kinetics had been experimentally validated previously, such as SbHCT [103], SbCCR1 [107], SbCAD2 [106], SbCOMT [105], and SbCCoAOMT [104]. Particularly, CCoAOMT catalyzes the methylation of caffeoyl-CoA to feruloyl-CoA, whereas COMT catalyzes the methylation of caffeic acid, 5-hydroxyconifer-aldehyde, or 5-hydroxyconifer-alcohol to facilitate S lignin production. Both COMT and CCoAOMT use *S*-adenosyl-l-methionine (SAM) as the methyl donor [104, 105]. In the RNA-seq data set from Rio/BTx406/R9188, three SAM synthases (SAMS) showed significantly higher expression levels in Rio than those in BTx406/R9188, while several genes required for SAM metabolism were highly expressed [108] (Additional file 13). Similarly, using our previously published metabolome results from the same samples for RNA-seq dataset1, we showed that SAM content was stable in Rio over the stages, but were undetected after the T1 stage in BTx406/R9188, indicating that Rio could have higher levels of SAM compared to BTx406/R9188 [12] (Additional files 4, 13). Overall, most of the monolignol biosynthetic genes continuously decreased when compared to the pre-anthesis and anthesis stages in BTx406/R9188, but in sweet genotypes, they were relatively stable at early post-anthesis stages after an initial decrease from pre-anthesis stage. This suggests that active monolignol biosynthesis is maintained at early stages of post-anthesis in sweet sorghum.

### Starch biosynthetic genes

Based on homologous and orthologous relationships between maize and sorghum genes, we identified genes encoding key enzymes in starch metabolism, including AGPase, SS, GBSS, SBE, ISA, GPT, GWD, PWD, and AMY (Additional file 12). We identified two AGPase small subunit genes and four AGPase large subunit genes, with Sobic.003G230500 and Sobic.007G101500 encoding the predictively cytoplasm-localized small and large subunits, respectively (Fig. 5). Interestingly, several AGPase subunits predicted to have plastidial localization (Sobic.002G160400, Sobic.009G245000, and Sobic.001G100000) were significantly upregulated in the sweet genotypes during sugar accumulation, but their expression levels were stable or decreased in BTx406/R9188. In contrast, the plastid-localized AGPase small subunits did not differ in expression trends between sorghum genotypes. Moreover, we identified two *GPT*s in sorghum (*SbGPT1*, Sobic.007G065500 and *SbGPT2*, Sobic.002G322000), of which the homologs in *Arabidopsis* function in G6P translocation into plastids and are responsible for providing G6P for the oxidative pentose phosphate pathway (OPPP) in specific tissues or fueling starch synthesis in non-green tissues, respectively [109, 110]. Expression of *SbGPT2* but not *SbGPT1* was upregulated and remained at high levels in sweet genotypes, but dramatically decreased in BTx406/R9188 (Fig. 5). Particularly, the Brix of introgression line R9188 can reach a high level comparable to Rio, but is not maintained and decreases at post-anthesis stages [12]. Among all the starch-related genes, *SbGPT2* is the only gene whose expression dynamics correlates well with soluble sugar levels at all stages [12]. Both *AGPase* and *GPT2* expression data indicated that ADP-glucose synthesis likely occurs in plastid and is highly active in sweet sorghum. Furthermore, several starch biosynthetic genes showed coordinated expression patterns like those observed in AGPase: (i) upregulation over the time course of stem sugar accumulation in sweet genotypes; (ii) significantly higher expression levels in Rio than in BTx406/R9188, and (iii) differential expression in Della and SIL05. They include two *SS* (Sobic.010G047700, Sobic.010G093400), one *GBSS* (Sobic.002G116000), two *ISA* (Sobic.007G204600, Sobic.009G127500), and two *SBE* genes (Sobic. 010G273800, Sobic.006G066800). The co-expression patterns between these starch biosynthetic genes are consistent with the notion that starch biosynthetic enzymes from multiple pathways form complexes in maize endosperm amyloplasts [56]. In addition, *SbGWD* (Sobic.010G143500) and *SbPWD* (Sobic.004G120100) with key roles in starch degradation also showed upregulated expression in sweet genotypes but not in BTx406/R9188. Similarly, a model of starch metabolism in Della has been proposed based on the expression of starch-metabolic genes from the Della RNA-seq data sets, and the model supports the activation of starch metabolism as sugar accumulates in sweet sorghum stem [11]. Taken together, the activation of starch-metabolic genes is associated with stem sugar levels and sink strength: all three sweet genotypes maintained high and upregulated expression of starch genes; R9188 with intermediate stem sugar [12] had lowered expression levels in some starch genes compared to sweet genotypes, including those encoding *SbGPT2*, *AGPase* (Sobic.001G100000, Sobic.007G101500), and *SS* (Sobic.010G047700, Sobic.010G093400).

### Sucrose-metabolic genes

**Fig. 6:**
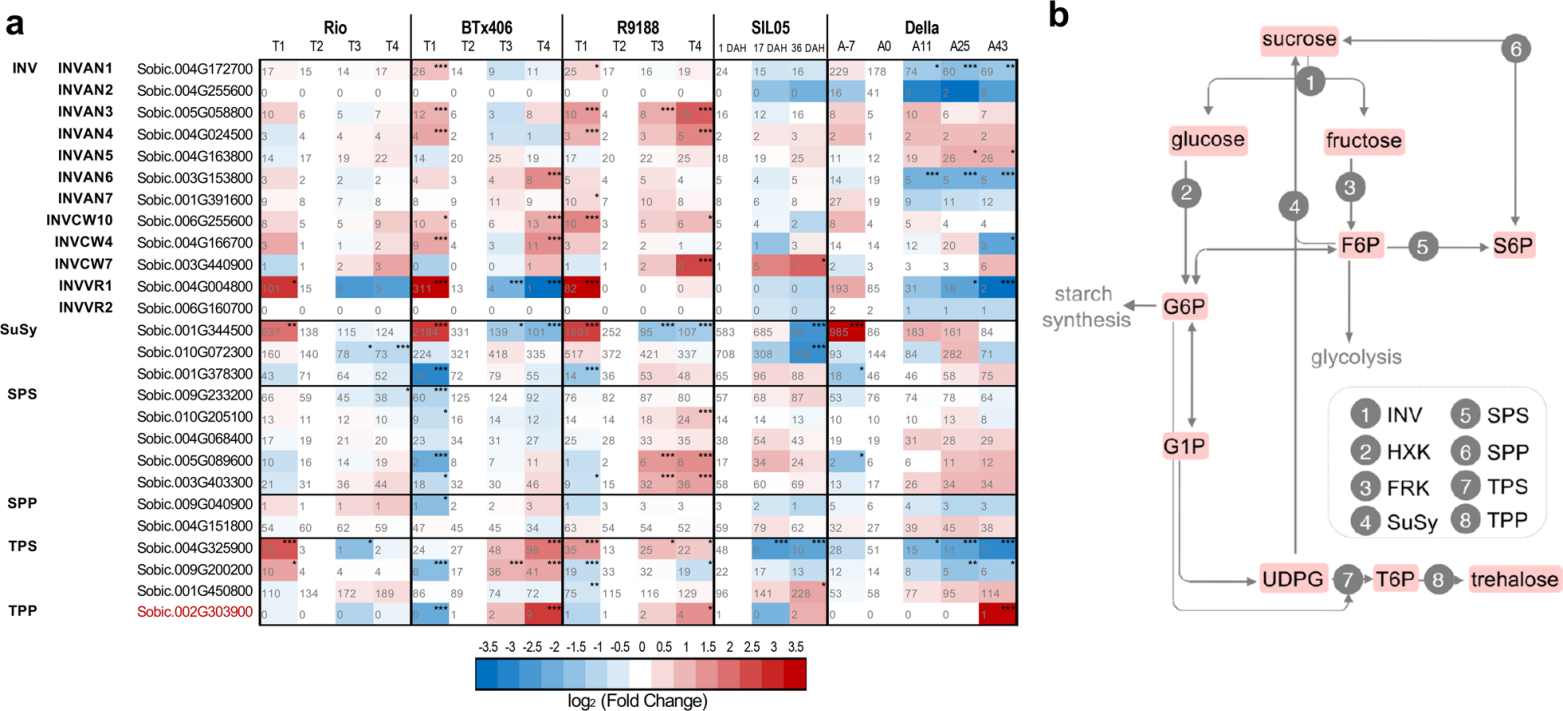
Comparison of sucrose-metabolic genes between sorghum genotypes. The expression dynamics of representative sucrose-metabolic genes were compared between sorghum genotypes and shown in heatmap (**a**), with the sucrose metabolism pathway shown in **b**. The cell colors are shaded to reflect the magnitude of log_2_ fold change of gene expression relative to the anthesis stages in each genotype. The expression levels in RPKM are labeled on each cell with statistical differences (*q* values determined by edgeR) indicated by asterisk (*q < 0.05; **q < 0.01; ***q < 0.005). The TPP gene highlighted in red was barely expressed in sweet lines Rio, Della and SIL05, but upregulated in BTx406 and R9188

Sucrose levels could be influenced by three sets of enzymes that are directly involved in channeling sucrose into primary metabolism: (i) invertase (INV) hydrolyzes sucrose into glucose and fructose; (ii) sucrose synthase (SuSy) hydrolyzes sucrose into fructose and UDP glucose; (iii) sucrose-phosphate synthase (SPS) and sucrose-phosphate phosphohydrolase (SPP) resynthesize sucrose (pathway in Fig. 6). The INVs are grouped into alkaline–neutral INVs (INVANs) and acid INVs based on their optimum pH, the latter being classified into cell wall INVs (INVCWs, insoluble) and vacuolar INVs (INVVRs, soluble). We identified 18 *INV*s in sorghum [111], including seven *INVAN*s, nine *INVCW*s, and two *INVVR*s, Additional file 14). We compared the *INV*s here with those identified in a sugarcane–sorghum comparative study [112] and found the *INV*s to be identical, with an additional *INVCW* (Sobic.001G099700) not expressed in the sorghum RNA-seq data sets. First, three *INVCW*s (Sobic.006G255600, Sobic.004G163800, and Sobic.003G440900) expressed in stems with varied expression patterns among genotypes, appeared not to be related to sugar accumulation (Fig. 6). Second, all seven *INVAN*s were expressed. Their expression patterns varied among genotypes and time points, but were not directly correlated to sucrose accumulation, indicating tightly regulated sucrose metabolism in cytosol. Third, the *INVVR* (Sobic.004G004800) with the highest expression levels among all *INV*s was sharply decreased at anthesis in all genotypes, a prerequisite for sugar accumulation in vacuole. Three *SuSy* genes were highly and differentially expressed: Sobic.001G344500 with the highest expression level was remarkably downregulated in all the genotypes, correlating with decrease of sucrose cleavage activity in Rio during sugar accumulation [113]; expression dynamics of Sobic.010G072300 and Sobic.001G378300 differed between genotypes. Several highly expressed SPSs and SPP (Sobic.004G151800) were stably detected and no clear trends were observed between sweet versus non-sweet genotypes. Furthermore, three *Trehalose 6*-*Phosphate Synthase* (*TPS*) and two *Trehalose Phosphate Phosphatase* (*TPP*) genes were highly transcribed. *TPS* and *TPP* genes jointly control the biosynthesis of trehalose 6-phosphate (T6P), an important signal and negative feedback regulator of sucrose levels [114]. One *TPP* gene (Sobic.002G303900) showed distinct expression patterns in sweet versus non-sweet sorghum. It was barely expressed in sweet genotypes but upregulated in Btx406/R9188 [12]. Functional studies are needed to elucidate their potential roles in carbon metabolism. It is worth noting that none of these sucrose-metabolic genes, except for *TPP*, showed upregulation when comparing sweet versus non-sweet genotypes, unlike those observed in cell wall and starch-metabolic genes (Figs. 4, 5, 6), suggesting two possibilities: sucrose metabolism is not a major limiting factor for stem sugar accumulation, or the regulatory role of sucrose-metabolic pathway lies in post-transcriptional levels. Taken together, the expression of *SPS*s, *SPP*s, *SuSy*s and seven intracellular *INVAN*s suggest active sucrose metabolism in stems, supporting previous conclusion that sucrose may be inverted and re-synthesized, while a significant portion was not metabolized during accumulation [40, 41].

### Sucrose transporters

**Fig. 7:**
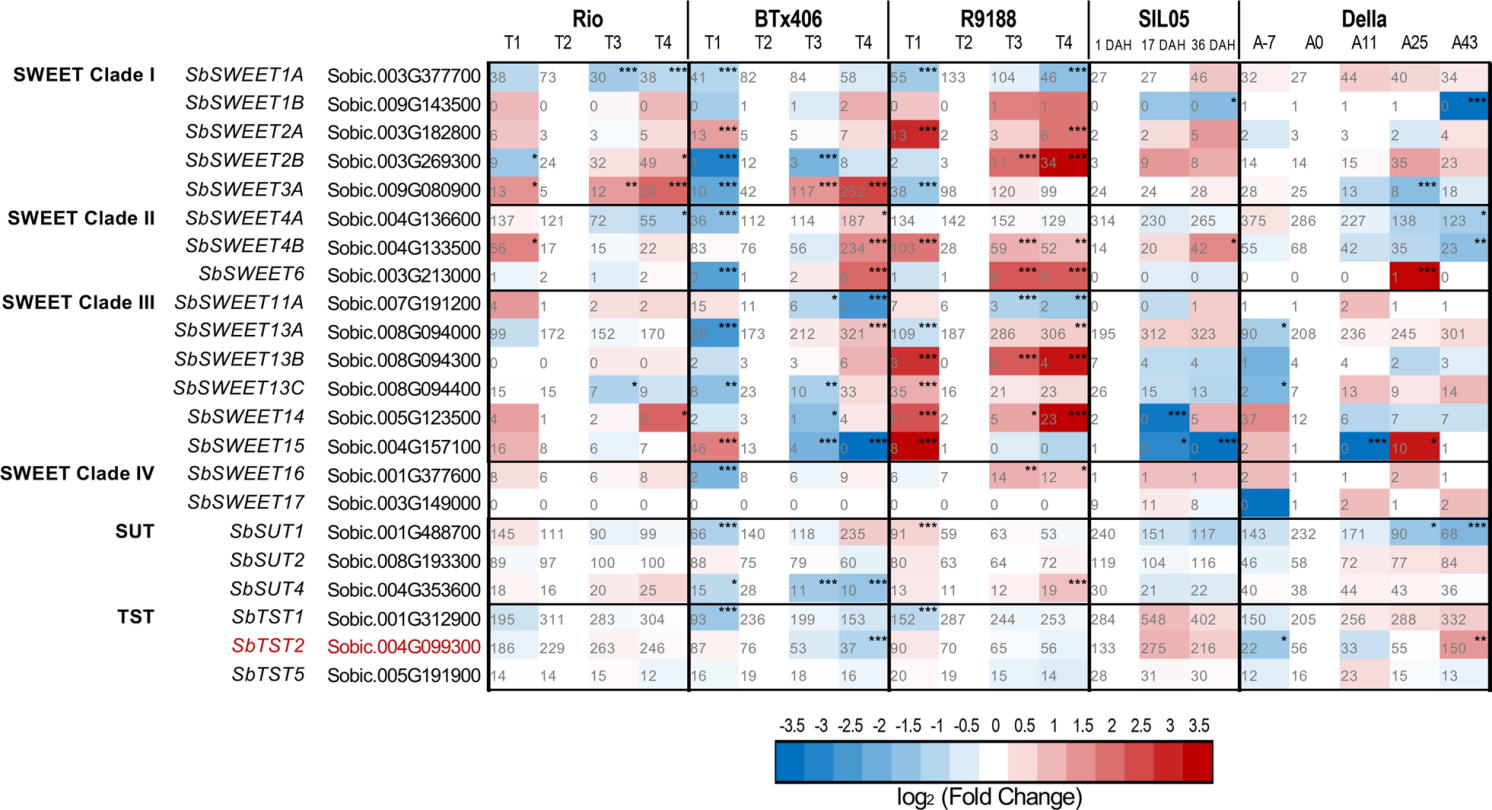
Comparison of sugar transporter genes between sorghum genotypes. The expression dynamics of SWEETs, SUTs, and TSTs were compared between sorghum genotypes and shown in heatmap. The cell colors are shaded to reflect the magnitude of log_2_ fold change of gene expression relative to the anthesis stages in each genotype. The expression levels in RPKM are labeled on each cell with statistical differences (*q* values determined by edgeR) indicated by asterisk (*q < 0.05; **q < 0.01; ***q < 0.005). *SbTST2* is highlighted in red, because upregulation of *SbTST2* was observed during post-anthesis stages in sweet lines Rio, Della, and SIL05, and its expression level in Rio was higher than those in non-sweet BTx406/R9188 (*p *< 0.05, determined by two-way ANOVA followed by multiple comparison)

To identify candidate sucrose transporters responsible for stem sugar accumulation, we analyzed three families: SUTs, TSTs, and SWEETs. A comprehensive phylogenetic analysis of SWEETs from all the sequenced species within Angiosperms defines four clades [47]. Results of SWEETs substrate selectivity have been reported in several species, such as *Arabidopsis* [48–50], rice [54], maize [52, 53], cucumber [51], and other species [115]. Published data suggest that the SWEET substrate selectivity correlates well with its phylogenetic clade (Additional file 15), indicating that only clade III SWEETs are likely to transport sucrose. However, the physiological functions of SWEETs remain to be characterized individually, which could be determined by their spatio-temporal expression patterns and/or the metabolic pathway that SWEET-mediated transport process could affect. According to our bioinformatics search, we identified 23 *SWEET*s and validated them to group into the four previously defined clades (Additional file 16) [47], with the designation of 20 *SbSWEET*s being consistent with the previous report [46]. By contrast, Mizuno et al. [45] identified 23 *SWEET*s including Sobic.003G149000, Sobic.003G038700, and Sobic.003G038800. Sobic.003G149000 contains two complete MtN3 domains and is designated *SbSWEET17* (Additional files 6, 7, 16). Sobic.003G038700 is grouped with clade II *SWEET*s and has two complete MtN3 domains, but Sobic.003G038800 has not, which is probably a tandemly duplicated copy of Sobic.003G038700 (Additional files 6, 7). These two putative *SWEET*s specifically expressed in the inflorescence at relatively lower levels compared to other *SWEET*s in the expression database MOROKOSHI (Additional file 7) [45, 84]. We focused on clade III *SWEET*s that could transport sucrose. Among the six differentially expressed clade III *SbSWEET*s, *SbSWEET13A* (Sobic.008094000) exhibited highest expression level in stems and was upregulated in all the genotypes (Fig. 7) [45]. Previously, *SbSWEET13A* was not found to be differentially expressed when compared between two grain and sweet sorghum lines [46]. Here, integration of several data sets demonstrated that *SbSWEET13A* was upregulated in stem tissues from all the investigated genotypes, probably excluding its role in determining sugar accumulation difference between grain and sweet sorghum (Fig. 7). Interestingly, the tandem-duplicated *SbSWEET13A/B/C* showed different expression levels and spatio-temporal specific patterns according to our data and other databases (Fig. 7). *SbSWEET13A/B* were preferentially expressed in green tissues, while *SbSWEET13C* was also expressed in roots, suggesting their functional divergence. *SbSWEET11A* exhibited lower expression levels in sweet genotypes than in the non-sweet genotypes, although its expression level was relatively lower than other clade III *SWEET*s. It is possible that *SbSWEET11A* might be highly expressed at particular cell types in stem tissue, leading to its expression level being diluted when measured in whole tissues. In addition, *SbSWEET13A* and *SbSWEET11A* appeared to be positively and negatively correlated to internode maturity or stages in non-sweet genotypes, but such a correlation was not observed in sweet genotypes, the biological significance of which needs further functional studies. The other clade III *SbSWEET* genes failed to generate a clear expression trends between sweet and non-sweet genotypes (Fig. 7), suggesting that SWEET-mediated sucrose transport may not be a major difference between sweet and non-sweet genotypes. Mizuno et al. [45] proposed that SbSWEET13A and 3A are involved in sucrose efflux from leaf; however, SbSWEET3A was grouped into clade I and homologous to other clade I SWEET3s from rice and maize (Fig. 7, Additional file 6) [52–54]. Similarly, the SbSWEET13A homolog in sugarcane (SsSWEET13C) was highly expressed in leaf mature zone and internode sclerenchyma cells, but expressed at very low levels in stem parenchyma, supporting a proposed role of SWEET13 in sucrose efflux in leaves [116]. SbSWEET4A, 4B, and 4C were suggested as candidates for sucrose transportation in panicle and stem, respectively, due to their spatial expression preference [45]. Here, SbSWEET4A/B/C are grouped into clade II together with their maize orthologs, consistent with the previous SWEET phylogenetic analysis [47]. Thus, the proposed roles of SbSWEET4s would be questioned because of their phylogeny–function correlation. It should be noted that the SWEET phylogenetic tree here resembles the one that covered all the sequenced species of Angiosperms, but differs from the tree reported in sorghum [45, 47]. This discrepancy may be due to the following differences in phylogenetic analysis: (i) species used for analysis; (ii) methods for amino acid sequence alignment; (iii) parameters for constructing phylogenetic tree.

Additionally, we identified three *SUT*s that were differentially expressed in at least one genotype (Fig. 7). Among the two highly expressed *SUT*s (*SbSUT1*, Sobic.001G488700; *SbSUT2*, Sobic.008G193300), only *SbSUT2* had a slightly higher expression level in sweet genotypes compared to BTx406/R9188 during post-anthesis, suggesting it as a candidate transporter related to stem sugar accumulation. The expression profiles of *SbTST*s showed that Sobic.001G312900 and Sobic.004G099300 also had higher expression levels (Fig. 7); they are homologous to the *TST1* and *TST2* in *Arabidopsis* [117] and sugar beet [118]. TST2 is the only TST member that can transport sucrose and is related to sucrose storage in vacuoles, which is confirmed in several species, including sugar beet [118], melon [119], and watermelon [120]. Here, *SbTST2* was highly expressed in sweet genotypes Rio and SIL-05, but had lower expression in BTx406/R9188 (two-way ANOVA, *p *< 0.05) and downregulation in BTx406 (Fig. 7). In Della, *SbTST2* also showed a gradual increase in expression levels, particularly at A43 and A67 stages (Fig. 7; Additional file 12). The discrepancy between *SbTST2* expression and sugar accumulation in Della could suggest post-translational regulation of its activity [121]. Overall, comparative expression analysis of sucrose transporters highlights the candidates that are likely be involved in sucrose transport and accumulation in sorghum stems, showing that *SbTST2*, but not SWEETs, is the top candidate for functional study and genetic improvement [42, 46]. Further investigations are needed to elucidate the physiological roles of these transporters in sorghum.

## Discussion

### Common metabolic network contributing to carbon sink strength in sweet sorghum internodes

The purposes of this study were to address whether candidate pathways/genes are specific for a given sweet sorghum genotype and to identify common expression features of primary metabolism and sugar transportation between different sweet sorghum genotypes. We, therefore, compared the dynamic transcriptomes of sugar-accumulating internodes between three unrelated sweet sorghum genotypes, with an emphasis on genes functioning in carbon utilization and sugar transport.

Previous studies have focused on sugar transporters SUTs, SWEETs, and TSTs that may contribute to stem sugar accumulation in sorghum. Analysis of SUTs expression in several tissues from grain sorghum BTx623 and sweet sorghum Rio at vegetative and anthesis stages showed variation of *SUT5* and *SUT6* but not of the highly expressed *SUT1* and *SUT2* [44]. Bihmidine et al. [42] compared *SUT* expression between grain and sweet sorghum and concluded that *SUT*s are unlikely to account for stem sugar in sorghum, because they showed differential expression in leaf tissue but not in stem tissue. The results of *SUT* expression in SIL05 revealed that their expression varied spatio-temporally but not more than twofold in the stem [45]. Our group characterized transcriptome dynamics over the period of sugar accumulation and the *SUTs’* results are consistent with the previous studies: (1) *SUT1*, *2* and *4* are highly expressed, while variation of expression exists between members (*SUT1* > *SUT2 *> *SUT4* at expression levels; Fig. 7); (2) variety-specific difference in expression levels were only observed for *SUT1* and *SUT2* at certain time points in the stem; (3) generally, the expression dynamics over time points and their comparison between several studied genotypes do not support roles in determining stem sugar difference between sorghum types. It is worth to note that the very low-expression levels of *SUT5* and *6* could deny their biological significance as transporters, and also makes it hard to ascertain differential expression due to high variance. Still, we could not exclude the possibility that *SUT5* and *SUT6* may be highly expressed at particular cell types in stem, similar to the above-mentioned case for *SbSWEET11A*. More recently, the expression analysis of *SbTST*s suggests *SbTST2* as a candidate gene accounting for stem sugar difference due to its differential expression between a grain and a sweet sorghum line [46]. Five *TST*s were identified based on our annotation [12]. The integrated expression data here show that *TST1* and *TST2* are the top two highly expressed *TST*s followed by *TST5*. Similar to the previous study, we only observed differential expression for *TST2* between grain and sweet genotypes with *TST2* upregulated in all sweet genotypes (Fig. 7) [46]. Taken together, *SbSUT2* and *SbTST2*, but not clade III SWEETs, may be candidate transporters for sucrose transportation in the stem, and *SbTST2* could be directly responsible for sucrose transportation into storage vacuoles [42, 46].

Though a candidate gene directing vacuolar storage of sucrose has been proposed [46], important biological questions remain to be addressed, such as: what factors determine that more sucrose is available in sweet sorghum than in grain sorghum for vacuolar storage? To what extent does carbon metabolism contribute to sugar accumulation? To address these questions, a system and integrated approach is needed to provide a cohesive and comparative picture of stem carbohydrate metabolism between distinct types of sorghum. Previous studies indicated that sucrose in vacuoles, starch in plastids, and carbohydrates in cell walls constitute the three major stem carbon reserves of sweet sorghum [11, 26]. Unlike the previous RNA-seq studies using one genotype, the major novelty of this comparative analysis is that we sought to replicate the comparison between sweet versus non-sweet genotypes using the RNA-seq data sets from Della and SIL05 [12, 26, 45]. Thus, such a comparison identified the common expression features in several pathways of primary metabolism and sugar transportation that differed from those seen in non-sweet BTx406/R9188. They are: (i) genes for cellulose, pectin, and hemicellulose synthesis were upregulated or highly expressed in sweet genotypes, but decreased in non-sweet lines; (ii) genes for phenylpropanoid and monolignol synthesis were decreased slower in sweet genotypes than in non-sweet lines; (iii) genes for starch metabolism were upregulated in sweet genotypes; (iv) when multiple sweet sorghum genotypes were investigated, the previous indications of INV or SWEETs as candidate genes for stem sugar accumulation were not strongly supported [26, 45]. Our comparison supports SbTST2 as a top candidate for functional studies and also identifies TPP as a candidate gene. While the transcriptional dynamics of cell wall and starch biosynthesis has been reported in Della, the association between cell wall/starch biosynthesis and stem sugar accumulation is established in the current study through comparative RNA-seq analysis. Such an association suggests that carbon allocation in stem might be coordinated among the carbon metabolic pathways (see below). In addition, the expression of 16 candidate genes highlighted in our analysis was determined by qRT-PCR. The RNA-seq and qPCR expression patterns of these 16 genes in Rio, BTx406, and R9188 were highly similar (Additional file 17).

The manners in which these metabolic pathways contribute to carbon utilization are different. Monolignol pathways are generally decreased from pre-anthesis stage, but the extent or rate of such decrease is less in sweet genotypes than in the non-sweet (Fig. 5). Expression of several key genes involved in primary cell wall components (cellulose, pectin and MLG) remained stable at early post-anthesis stages in sweet sorghum, but was gradually reduced in non-sweet genotypes (Fig. 4). By contrast, expression levels of many starch synthetic genes were well correlated with stem sugar accumulation, SbGPT2 appearing to be a limiting factor for starch due to its potential role of providing G6P (Fig. 7). Close examination of these metabolic pathways highlights several candidate genes (e.g., *AGPase*, *GPT2*, and *CesA*) that may be used for regulating stem sugar accumulation through up- or downregulation of specific pathways. Additionally, we examined the *D* gene expression (Sobic.006G147400), because it is a major regulator of stem juiciness and aerenchyma formation [30–33]. Results showed that the *D* gene determined stem juiciness, but did not affect the sugar concentration in juice [31]. Indeed, the *D* gene was not expressed in Rio, R9188, Della, and SIL05, with very low-expression detected in BTx406 (RPKM ~ 2; Additional file 12), consistent with the previous results that a nonfunctional *D* gene is required for juicy stem, the prerequisite of sweet sorghum [30–33].

Although more research will be needed for further functional studies, the results allow us to propose a model for carbon allocation in sink stems of sweet sorghum (Additional file 18). In this model, sucrose transported from long distance could flow into four metabolic fates in storage parenchyma cells: first, sucrose inversion and re-synthesis for consuming energy could provide materials for starch and cell wall synthesis (glucose-6-phosphate and UDPG, respectively); second, vacuolar storage of sucrose could serve not only as a carbon reserve, but also as a temporary pool for starch and cell wall metabolism, when sucrose supply fluctuates; third, starch synthesis and storage in plastids could flow from degradative products of sucrose; fourth, carbohydrates could be used for cellulose synthesis for primary cell wall and maintaining relatively active monolignol biosynthesis compared to grain sorghum. This carbon sink model represents carbon allocation in several new ways compared with the previous studies. (1) It provides transcriptomics evidence supporting the notion that carbon allocation within sorghum sink stem is likely to be coordinated by different carbon utilization routes and this is likely a common feature of the sweet sorghum stems. (2) It highlights and summarizes reliable candidate genes for modulating stem carbon compositions from previous identified pathways. (3) It provides transcriptomics evidence indicating that sucrose cleavage and re-synthesis could occur in sorghum stem. (4) It clarifies the potential roles of SWEETs in leaf sucrose efflux but not in the stem sugar difference between the sweet versus non-sweet genotypes.

The comparative transcriptome approach used herein has three advantages. (i) Integration of the three data sets: to overcome the limitations in differential expression analysis for data sets 2 and 3, the biological variance from similar tissues and time points (dataset1) was employed to identify differentially expressed genes in Della and SIL05 (“Methods”; Additional file 4); besides, differential expression analysis within each dataset allowed the association between expression dynamics with sugar accumulation but avoided potential problems raised from direct comparison of the genotypes between datasets, such as removal of the batch effects between datasets, intrinsic expression difference between genotypes unrelated to sugar. (ii) We consider genes involved in the three major carbohydrate reserves that are supported by previous phenotype results [11, 12, 26] and, therefore, provide the metabolism-related transcriptome dynamics at genome-wide level. (iii) The proposed model based on our comparative analyses not only provides promising candidate genes for bioenergy improvement, but also may serve as a guidance for understanding and manipulating carbon composition of sorghum stem at transcriptional level. In line with the notion of coordination between carbon utilization routes, this model suggests that it is possible to modulate stem biomass composition through up- or downregulation of specific primary metabolic pathways. Similar coordination between sucrose and starch metabolism has been well demonstrated and applied in the case of various sweet corns, which are caused by mutations in starch biosynthetic genes [122]. Generally, several mutants in starch defects result in increased sucrose content in maize kernels, representing the re-distribution of carbon from starch synthesis to sugar metabolism. Applying a similar concept in sorghum, stem starch biosynthesis might be knocked down to enhance sucrose accumulation in vacuoles via repression of *GPT2* or key starch biosynthetic enzymes. Sucrose storage in vacuoles might be also enhanced by overexpressing *SbTST2* [46]. Also, it might be possible to regulate the primary metabolic pathways downstream of T6P signal by modifying T6P content via transgenic *SbTPP*s. A similar approach of heterologous expression of *TPP* has been shown to be efficient in altering metabolism in maize endosperm [123, 124]. Additionally, stem biomass composition results from the sorghum mutants in monolignol biosynthetic could also be explained this way. Near isogenic lines (NIL) carrying the *bmr6* mutation in grain sorghum background showed significantly increased total free soluble sugars, whereas the effect of *bmr12* mutant varied depending on genetic backgrounds [125]. Decreased lignin contents are associated with slightly, though statistically not significant, increase in stem sugar concentration, [126].

Still, further improvement of the current model will be required. Particularly, functional validation of the candidate genes in sorghum is necessary to improve our understanding of the metabolic consequences of carbon utilization. On the other hand, incorporation of metabolomics and proteomics data in the future will refine the model, as transcriptomics data themselves have limitations in interpreting metabolism due to multiple layers of regulation at post-transcriptional, protein and metabolite levels [127, 128]. Also, our interpretations may come with caveats based on the possibility that the expression data analyzed may not be truly representative in some instances, since some of the data sets are not replicated. Moreover, several cell wall components, such as xylan and glucan, account for considerable fractions of carbon utilization [26] are not included in the present model due to missing information on their metabolic genes in sorghum and closely related species.

## Conclusions

Here, we have presented the first comparative transcriptome analysis of sugar-accumulating internodes in sorghum that is relevant to bioenergy research at a gene discovery level. The common transcriptome features indicate differences in several primary metabolic pathways between the sweet and non-sweet sorghums, suggesting the metabolic networks possibly coordinating carbon allocation and sink strength in the sorghum internode. Specifically, several genes, including those involved in cellulose and monolignol synthesis (*CesA*, *PTAL,* and *CCR*), starch metabolism (*AGPase*, *SS*, *SBE* and G6P-translocator *SbGPT2*), and sucrose metabolism and transportation (*TPP* and *TST2*), were strongly correlated with the three sweet sorghum genotypes compared to the non-sweet lines, serving as candidates for functional studies of carbon manipulation in sorghum stem. This study also shows that a combination of multiple advanced resources (including metabolites, expression data sets, genotypes, and conditions of sorghum stem sink) provides a comprehensive and cohesive picture of the complexity of carbon sink strength in sorghum stem, which might not be achieved by a single data set. The many candidate genes identified here could be manipulated and studied to further our understanding and utilization of carbon allocation and/or sugar accumulation in bioenergy crops.

## Supplementary information

**Additional file 1.** Pedigrees of sweet sorghum Rio, Della and SIL-05.

**Additional file 2.** Information of the RNA-seq samples used in this study.

**Additional file 3.** Variation in expression of the reference genes in sorghum before and after removal of batch effect.

**Additional file 4.** Supplementary methods.

**Additional file 5.** Annotation of genes involved in the primary metabolic pathways.

**Additional file 6.** Annotation and nomenclature of SbSWEETs.

**Additional file 7.** Spatio-temporal expression patterns of sorghum SWEETs.

**Additional file 8.** Primers used for real-time quantitative PCR (qPCR).

**Additional file 9.** Dynamics of internode Brix (**a**) and water concentration (**b**) over stem sugar accumulation.

**Additional file 10.** Distribution of the expression levels for all the RNA-seq data sets used before (a) and after (b) batch-effect removal.

**Additional file 11.** Hierarchical clustering (HC) of the RNA-seq data sets used.

**Additional file 12.** Selected differentially expressed genes in the cellulose biosynthesis, monolignol biosynthesis and starch and sucrose metabolic pathways.

**Additional file 13.** The dynamics of tyrosine and SAM and the expression levels of genes involved in SAM metabolism.

**Additional file 14.** Annotation of sorghum invertases (INVs) and their homologs and orthologs in maize.

**Additional file 15.** Summary of the substrate selectivity and phylogenetic clades of SWEET proteins.

**Additional file 16.** The phylogenetic tree of SWEET proteins from rice, maize and sorghum.

**Additional file 17.** Validation of RNA-seq results by qPCR.

**Additional file 18.** A proposed model for carbon partitioning and sink strength in sorghum internodes.

## Data Availability

All data generated or analyzed during this study are included in this published article and its additional information files.

## Abbreviations

ADPG: ADP-glucose
AGPase: ADP-glucose pyrophosphorylase
BAM: β-amylase
bmr: brown midrib
C3H: 4-coumarate 3-hydroxylase
C4H: cinnamate 4-hydroxylase
CAD: cinnamyl alcohol dehydrogenase
CCoAOMT: caffeoyl-coenzyme A 3-*O*-methyltransferase
COMT: caffeic acid *O*-methyltransferase
CCR: cinnamyl-CoA reductase
CesA: cellulose synthase
Csl: cellulose synthase-like
DAF: days to flowering
DAH: days to heading
DBE: starch debranching enzyme
DEG: differentially expressed gene
DPE: disproportionating enzyme
F5H: ferulate 5-hydroxylase
G1P: glucose-1-phosphate
G6P: glucose-6-phosphate
GBSS: granule-bound starch synthase
GPT: glucose-6-phosphate translocator
GWAS: genome-wide association analysis
GWD: glucan–water dikinase
HC: hierarchical clustering
HCT: hydroxycinnamoyltransferase
INV: invertase
INVAN: alkaline–neutral invertase
INVCW: cell wall invertase
INVVR: vacuolar invertase
ISA: isoamylase
PAL: phenylalanine ammonia-lyase
PCA: principle component analysis
PCW: primary cell wall
PGM: phosphoglucomutase
PHT: plant height
PTAL: l-Phe/-Tyr l-Phe/-Tyr ammonia-lyase
PWD: phosphor-glucan–water dikinase
QTL: quantitative trait loci
RPKM: reads per kilobase of exon per million mapped sequence reads
SAM: *S*-adenosyl-l-methionine
SBE: starch branching enzyme
SCW: secondary cell wall
SPP: sucrose-phosphate phosphohydrolase
SPS: sucrose-phosphate synthase
SS: soluble starch synthase
SuSy: sucrose synthase
SUT: sucrose transporter
SWEET: Sugars Will Eventually be Exported Transporters
T6P: trehalose-6-phosphate
TPP: trehalose-6-phosphate phosphatase
TPS: trehalose-6-phosphate synthase
TST: Tonoplast Sugar Transporter
USDA-NPGS: United States Department of Agriculture National Plant Germplasm System
4CL: 4-coumarate:CoA ligase
