# Correction to: Common metabolic networks contribute to carbon sink strength of sorghum internodes: implications for bioenergy improvement

**DOI:** 10.1186/s13068-019-1621-6

**Published:** 2019-12-11

**Authors:** Yin Li, Min Tu, Yaping Feng, Wenqin Wang, Joachim Messing

**Affiliations:** 1 Waksman Institute of Microbiology, Rutgers, The State University of New Jersey, Piscataway, NJ 08854 USA; 2 School of Agriculture and Biology, Shanghai Jiao Tong University, 800 Dong Chuan Road, Shanghai, 200240 China

## Correction to: Biotechnol Biofuels (2019) 12:274 10.1186/s13068-019-1612-7

The original version of the article [1] unfortunately contained a mistake in author’s first name. The name of the author has been corrected from Wenqing Wang to Wenqin Wang in this correction article. The original article [1] has been corrected.
