# Supplementary material for: Common metabolic networks contribute to carbon sink strength of sorghum internodes: implications for bioenergy improvement
Source: Biotechnol Biofuels. 2019 Nov 20;12:274. doi: 10.1186/s13068-019-1612-7 (PMC6868837; doi:10.1186/s13068-019-1612-7)
Supplement: Supplementary file 1 — Additional file 1. Pedigrees of sweet sorghum Rio, Della and SIL-05. [file 13068_2019_1612_MOESM1_ESM.docx]

**Additional Files for the manuscript:**

**Common metabolic networks contributing to carbon sink strength of sorghum internodes and implications for bioenergy improvement**

Yin Li^1^, Min Tu^1^, Yaping Feng^1^, Wenqing Wang^2^, Joachim Messing^1,*^

^1^Waksman Institute of Microbiology, Rutgers, The State University of New Jersey, Piscataway, NJ 08854, USA.

^2^School of Agriculture and Biology, Shanghai Jiaotong University, 800 Dong Chuan Road, Shanghai 200240, China.

* Correspondence: messing@waksman.rutgers.edu

**
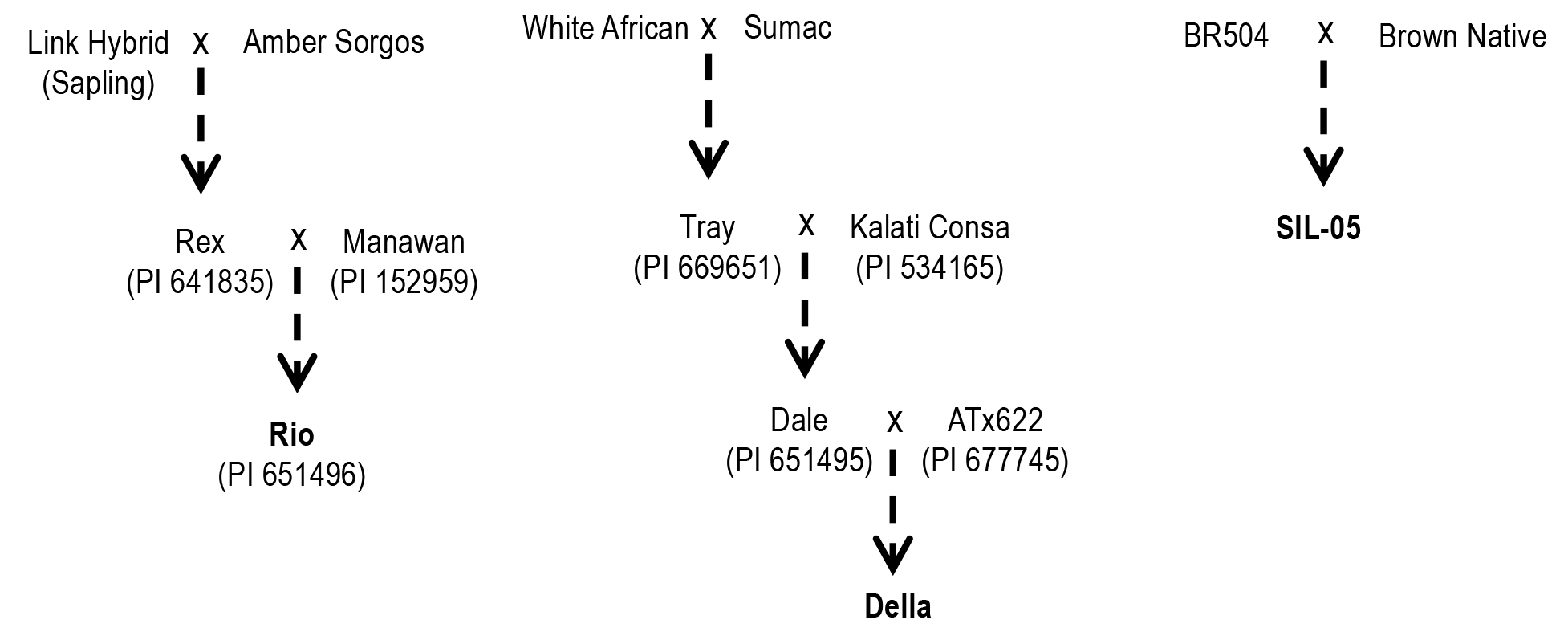
**

**Additional file 1.** Pedigrees of sweet sorghum Rio, Della and SIL-05.
