# Supplementary material for: Common metabolic networks contribute to carbon sink strength of sorghum internodes: implications for bioenergy improvement
Source: Biotechnol Biofuels. 2019 Nov 20;12:274. doi: 10.1186/s13068-019-1612-7 (PMC6868837; doi:10.1186/s13068-019-1612-7)
Supplement: Supplementary file 3 — Additional file 3. Variation in expression of the reference genes in sorghum before and after removal of batch effect. [file 13068_2019_1612_MOESM3_ESM.docx]

**Additional file 3.** Variation in expression of the reference genes in sorghum before and after removal of batch effect.

| **Gene Annotation** | **geneID** | **References** | **Before normalization** | | |  | **After normalization** | |  |  |
| --- | --- | --- | --- | --- | --- | --- | --- | --- | --- | --- |
|  |  |  | **CV_data1** | **CV_data2** | **CV_data3** | **CV_allData** | **CV_data1** | **CV_data2** | **CV_data3** | **CV_allData** |
| ubiquitin | Sobic.001G136900 | Abdel-Ghny SE et al. 2016 | 0.258 | 0.244 | 0.098 | 0.261 | 0.246 | 0.210 | 0.107 | 0.229 |
| ACP2 [Acyl carrier protein2] | Sobic.002G280400 | Reddy PS et al. 2016 | 0.196 | 0.128 | 0.432 | 0.252 | 0.185 | 0.132 | 0.455 | 0.238 |
| EIF 4a [Eukaryotic Initiation factor 4A] | Sobic.004G039400 | Reddy PS et al. 2016 | 0.292 | 0.249 | 0.394 | 0.341 | 0.305 | 0.280 | 0.398 | 0.326 |
| PP2A [Serine/threonine- protein Phosphatase] | Sobic.004G092500 | Reddy PS et al. 2016 | 0.154 | 0.090 | 0.221 | 0.168 | 0.153 | 0.082 | 0.171 | 0.151 |
| Ubiquitin (protein1 isoformC) | Sobic.004G273300 | Reddy PS et al. 2016 | 0.237 | 0.181 | 0.220 | 0.284 | 0.245 | 0.162 | 0.209 | 0.274 |
| 6PGDH [6-phosphogluconate dehydrogenase] | Sobic.005G115600 | Reddy PS et al. 2016 | 0.414 | 0.491 | 0.406 | 0.520 | 0.411 | 0.439 | 0.352 | 0.500 |
| ACTIN | Sobic.008G047000 | Li et al. 2014 | 0.273 | 0.464 | 0.470 | 0.351 | 0.293 | 0.378 | 0.377 | 0.346 |
| ACTIN | Sobic.009G005900 | Li et al. 2014 | 0.187 | 0.286 | 0.375 | 0.270 | 0.203 | 0.241 | 0.363 | 0.253 |
| EF1a | Sobic.010G182200 | Milne RJ et al. 2013 | 0.202 | 0.263 | 0.370 | 0.297 | 0.213 | 0.244 | 0.383 | 0.281 |
| EF1a | Sobic.010G182600 | Milne RJ et al. 2013 | 0.355 | 0.189 | 0.474 | 0.395 | 0.309 | 0.252 | 0.480 | 0.372 |

Note: CV= coefficient of variance; Data1 = RIO/R9188/BTx406 RNA-seq dataset; Data2 = Della RNA-seq dataset; Data3 = SIL-05 RNA-seq dataset; References where these genes were used as reference genes for qRT--PCR in sorghum are given as below.

**References for Additional file 3:**

- Abdel-Ghany SE, Hamilton M, Jacobi JL, Ngam P, Devitt N, Schilkey F, Ben-Hur A, Reddy ASN. 2016. A survey of the sorghum transcriptome usingcsingle-molecule long reads. Nature Communications, 7, 11706.
- Reddy PS, Reddy DS, Sivasakthi K, Bhatnagar-Mathur P, Vadez V, Sharma KK. 2016. Evaluation of Sorghum [Sorghumbicolor(L.)] Reference Genes in Various Tissues and under Abiotic Stress Conditions for Quantitative Real-Time PCR Data Normalization. Frontiers in Plant Science, 7, 529.
- Li X, Su M, Li X, Cheng L, Qi D, Chen S, Liu G. 2014. Molecular characterization and expression patterns of sucrosetransport-related genes in sweet sorghum under defoliation. Acta Physiol. Plant, 36, 1251-1259.
- Milne RJ, Byrt CS, Patrick JW, Grof CP. 2013. Are sucrose transporter expression profiles linked with patterns of biomass partitioning in Sorghum phenotypes? Frontiers in Plant Science, 4, 223.
