# Supplementary material for: Common metabolic networks contribute to carbon sink strength of sorghum internodes: implications for bioenergy improvement
Source: Biotechnol Biofuels. 2019 Nov 20;12:274. doi: 10.1186/s13068-019-1612-7 (PMC6868837; doi:10.1186/s13068-019-1612-7)
Supplement: Supplementary file 4 — Additional file 4. Supplementary methods. [file 13068_2019_1612_MOESM4_ESM.docx]

**Additional file 4.** Supplementary methods for the identification of genes involved in primary metabolism and sugar transport.

**
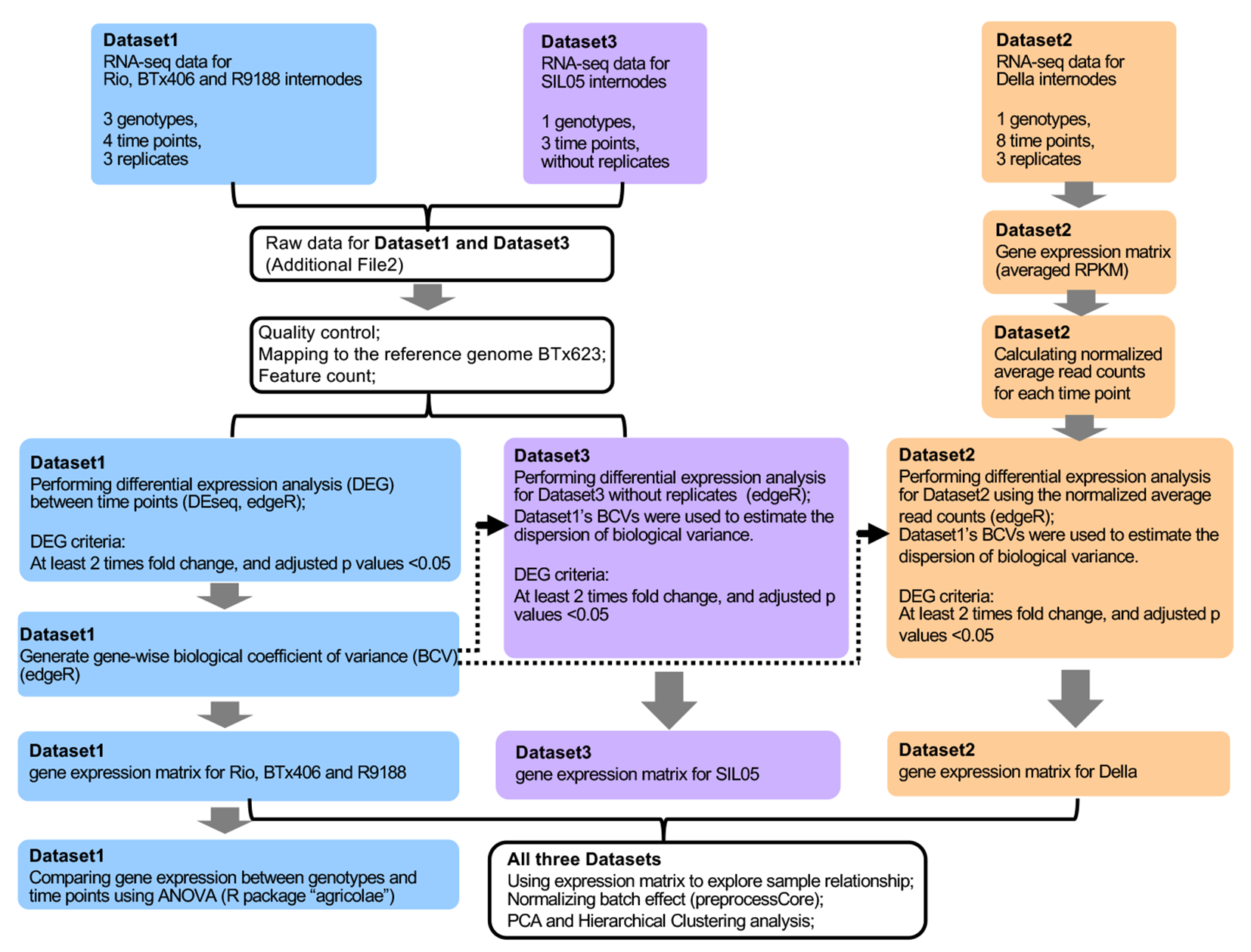
**

**Batch effect normalization for expression matrix**

To normalize potential batch effects between the three RNA-seq studies, gene expression data using RPKM values were prepared. Expression datasets using RPKM values for the first and second RNA-seq experiments were reported previously. For the third RNA-seq data set, raw reads were obtained and subjected to quality check according to Mizuno *et al.* [51]. Filtered reads were mapped to the sorghum reference genome (BTx623, Sbicolor_v2.1_255) using TopHat v2.0.14 (a maximum mismatch of 9 bp and default settings for other parameters) [13,73]. Read counts were calculated using ‘HTseq’ with uniquely-mapped reads and RPKM values were calculated for SIL-05 [74]. All the three datasets were normalized using RPKM. Genes with RPKM values ≥1 were considered as expressed genes. The distribution of gene expression levels (log2-transformed RPKM+1) were compared (Fig. S2a). Quantile normalization (calculated by the R package “preprocessCore”) was used to remove batch effects between the three datasets [75-77], resulting in similar distribution of gene expression levels between RNA-seq samples (Fig. S2b). Sorghum reference genes for qRT-PCR had smaller coefficient of variance after removal of batch effects (Additional File 3).

**Identification of genes involved in primary metabolism and sugar transport**

***Genes involved in cell wall metabolism.***

Genes related to cell wall metabolism were identified according to Rai *et al.* (2016). Several gene families in phenylpropanoid and monolignol pathways were characterized in sorghum with phylogenetic and functional approaches, including CAD (Saballos *et al.*, 2009; Jun *et al.*, 2017), CCR (Barakat *et al.*, 2011; Sattler *et al.*, 2017), CCoAOMT (Walker *et al.*, 2016), COMT (Green *et al.*, 2014), HCT (Walker *et al.*, 2013), PAL (Jun *et al.*, 2018) and 4CL (Saballos *et al.*, 2012). Categorization of the cellulose synthase (*Csl*) gene superfamily, including several families of Csl-like families, was identified according to a systematic phylogenetic-functional study (Little *et al.* 2018). The functional mixed-linkage (1,3; 1,4) β-glucan (MLG) synthase of the CslF6 family was reported elsewhere (Dimitroff *et al.* 2016). Other families of cell wall-related genes were collected elsewhere (Carpita *et al.*, 2001).

***Genes involved in starch and sucrose metabolism, and glycolysis***

Different sources, including Plant Metabolic Network (PMN, Chae *et al.* 2014), KEGG (Kanehisa *et al.* 2015), MapMan (Usadel *et al.* 2009) and Campbell *et al.* (2016), were integrated for annotating genes related to starch, sucrose metabolism and some steps of glycolysis. To increase gene annotation accuracy, maize gene models with proteomics evidence were used (Friso *et al.* 2010) and translated to sorghum gene models based on their orthologous relationships (Zhang *et al.* 2017). GWD (Sobic.010G143500) and PWD (Sobic.004G120100) with major roles in starch degradation were identified based on their homologs in maize. Genes encoding invertase (INV) were identified based on a recent manual curation in maize (Juarez-Golunga *et al.* 2018) and compared with an INV family study in sugarcane (Wang et al. 2017). The trehalose-6-phosphate synthase (TPS) and trehalose-6-phosphate phosphatase (TPP) families were described previously (Li *et al.* 2019).

***Genes involved in sucrose transportation.***

Genes encoding SUTs, SWEETs and TSTs were evaluated previously (Li *et al.* 2018). All the gene annotation information is shown in Table S3.

**Metabolic profiling.**

The samples were collected from a field with split plot design with three replicated blocks at the Waksman Institute, Rutgers, The State University of New Jersey. Each block consisted of three plots, with each sorghum genotype randomly planted in one plot. Each plot consisted of five rows which had 25 plants per row. To obtain the dynamics of soluble sugars in sorghum stem, five time points were chosen to collect stem tissues for detailed analysis: flag leaf stage (T1), 100% flowering (T2), 10 days after flowering (T3), 15 days after flowering (T4) and 30 days after flowering (T5). In each plot, the three central rows were further divided into five subplots containing nine plants per subplot, with each subplot (nine plants) randomly corresponding to one harvest time point. the upper internodes (internode No. 2, 3 and 4) were harvested from the nine plants per subplot at each of the five time points. Internodes were numbered from top to bottom. When sampling the internodes, two sections in the middle of each internode were used for RNA-seq (dataset1 used in the present study) and metabolome analysis. The six plants in each subplot were pooled together as one biological sample used for metabolic profiling. In total, six biological replicates (two samples multiplied by three blocks) were used for untargeted metabolomics analysis. The metabolome analysis was performed by Metabolon, Inc. under a service contract with Syngenta. Tyrosine and S-adenosyl-L-methionine (SAM) were identified and quantified in Rio, BTx406 and R9188 with untargeted metabolomics. The untargeted metabolomics platform is composed of four independent platforms: UHPLC-MS/MS optimized for basic species, UHPLC-MS/MS optimized for acidic species, polar LC platform (UHPLC(HILIC)-MS/MS) and GC-MS (Evans et al., 2009; Ohta et al., 2009). Briefly, each sample was extracted in methanol with recovery standards using an automated MicroLab STAR system (Hamilton). The resulting extract was divided into five fractions, with four fractions subjected to the four platforms, respectively, and one fraction reserved for backup. For LC methods, the analysis was carried out by using a Water Acquity UHPLC coupled to a Thermo Scientific Q-Exactive mass spectrometer equipped with an electrospray ionization source and Orbitrap mass analyzer. For GC-MS, samples were derivatized using bis-trimethyl-silyl-triflouroacetamide and then analyzed on a Thermo-Finnigan Trace DSQ fast-scanning single-quadrupole mass spectrometer. Known chemicals were identified by comparison to Metabolon’s library entries of purified standard compounds based on retention time/index (RI), mass to charge ratio (m/z), and MS/MS spectral data. A full description of the untargeted metabolomics method can be found under Supplemental Information. For data visualization, the raw area counts for each compound were median scaled. Detailed methods for sample collection and metabolomics analysis had been published elsewhere (Li et al. 2019)

**References for Additional file 4:**

1. Rai KM, Thu SW, Balasubramanian VK, Cobos CJ, Disasa T, Mendu V. Identification, characterization, and expression analysis of cell wall related genes in *Sorghum bicolor* L. Moench, a food, fodder, and biofuel crop. Front Plant Sci. 2016; 7:1287.
2. Saballos A, Ejeta G, Sanchez E, Kang C, Vermerris W. A Genome wide analysis of the cinnamyl alcohol dehydrogenase family in sorghum [*Sorghum bicolor* (L.) Moench] identifies *SbCAD2* as the *brown midrib6* gene. Genetics. 2009; 181(2):783-795.
3. Jun SY, Walker AM, Kim H, Ralph J, Vermerris W, Sattler SE, Kang C. The enzyme activity and substrate specificity of two major cinnamyl alcohol dehydrogenases in sorghum *Sorghum bicolor.*, SbCAD2 and SbCAD4. Plant Physiol. 2017; 174(4):2128-2145.
4. Barakat A, Yassin NB, Park JS, Choi A, Herr J, Carlson JE. Comparative and phylogenomic analyses of cinnamoyl-CoA reductase and cinnamoyl-CoA-reductase-like gene family in land plants. Plant Sci. 2011; 181(3):249-257.
5. Sattler SA, Walker AM, Vermerris W, Sattler SE, Kang C. Structural and biochemical characterization of cinnamoyl-CoA reductases. Plant Physiol. 2017; 173(2):1031-1044.
6. Walker AM, Sattler SA, Regner M, Jones JP, Ralph J, Vermerris W, Sattler SE, Kang C. The structure and catalytic mechanism of *Sorghum bicolor* caffeoyl-CoA O-methyltransferase. Plant Physiol. 2016; 172(1):78-92.
7. Green AR, Lewis KM, Barr JT, Jones JP, Lu F, Ralph J, Vermerris W, Sattler SE, Kang C. Determination of the structure and catalytic mechanism of *Sorghum bicolor* caffeic acid O-methyltransferase and the structural impact of three brown midrib12 mutations. Plant Physiol. 2014; 165(4):1440-1456.
8. Walker AM, Hayes RP, Youn B, Vermerris W, Sattler SE, Kang C. Elucidation of the structure and reaction mechanism of Sorghum hydroxycinnamoyltransferase and its structural relationship to other coenzyme A-dependent transferases and synthases. Plant Physiol. 2013; 162(2):640-651.
9. Jun SY, Sattler SA, Cortez GS, Vermerris W, Sattler SE, Kang C. Biochemical and structural analysis of substrate specificity of a phenylalanine ammonia-lyase. Plant Physiol. 2018; 176(2):1452-1468.
10. Saballos A, Sattler SE, Sanchez E, Foster TP, Xin Z, Kang C, Pedersen JF, et al. *Brown midrib2* (*Bmr2*) encodes the major 4-coumarate: coenzyme A ligase involved in lignin biosynthesis in sorghum (*Sorghum bicolor* (L.) Moench). Plant J. 2012; 70(5):818-830.
11. Little A, Schwerdt JG, Shirley NJ, Khor SF, Neumann K, O’Donovan LA, Lahnstein J, Collins HM, Henderson M, Fincher GE, et al. Revised phylogeny of the cellulose synthase gene superfamily: insights into cell wall evolution. Plant Physiol. 2018; 177(3):1124-1141.
12. Dimitroff G, Little A, Lahnstein J, Schwerdt JG, Srivastava V, Bulone V, Burton RA, Fincher GB. (1,3;1,4)-β-Glucan biosynthesis by the CSLF6 enzyme: position and flexibility of catalytic residues influence product fine structure. Biochemistry. 2016; 55(16):2054-2061.
13. Carpita NC, Tierney M, Campbell M. Molecular biology of the plant cell wall: searching for the genes that define structure, architecture and dynamics. Plant Mol Biol. 2001; 47(1-2):1-5.
14. Chae L, Kim T, Nilo-Poyanco R, Rhee SY. Genomic signatures of specialized metabolism in plants. Science. 2014; 344(6183):510–513.
15. Kanehisa M, Sato Y, Kawashima M, Furumichi M, Tanabe M. KEGG as a reference resource for gene and protein annotation. Nucleic Acid Res. 2015; 44(D1):D457–D462.
16. Usadel B, Poree F, Nagel A, Lohse M, Czedik-Eysenberg A, Stitt M. A guide to using MapMan to visualize and compare omics data in plants: a case study in the crop species, Maize. Plant Cell Environ. 2009; 32(9):1211-1229.
17. Campbell BC, Gilding EK, Mace ES, Tai S, Tao Y, Prentis PJ, Thomelin P, et al. Domestication and the storage starch biosynthesis pathway: signatures of selection from a whole sorghum genome sequencing strategy. Plant Biotech J. 2016; 14(12):2240-2253.
18. Friso G, Majeran W, Huang M, Sun Q, J van Wijk K. Reconstruction of metabolic pathways, protein expression, and homeostasis machineries across maize bundle sheath and mesophyll chloroplasts: large-scale quantitative proteomics using the first maize genome assembly. Plant Physiol. 2010; 152(3):1219-1250.
19. Zhang Y, Ngu DW, Carvalho D, Liang Z, Qiu Y, Roston RL, Schnable JC. Differentially regulated orthologs in sorghum and the subgenomes of maize. Plant Cell. 2017; 29(8):1938-1951.
20. Juarez-Colunga S, Lopez-Gonzalez C, Morales-Elias NC, Massange-Sanchez JA, Trachsel S, Tiessen A. Genome-wide analysis of the invertase gene family from maize. Planta. 2018; 97(4-5):385-406.
21. Wang L, Zheng Y, Ding S, Zhang Q, Chen Y, Zhang J. Molecular cloning, structure, phylogeny and expression analysis of the invertase gene family in sugarcane. BMC Plant Biol. 2017; 17:109.
22. Li Y, Wang W, Feng Y, Tu M, Wittich PE, Bate NJ, Messing J. Transcriptome and metabolome reveal distinct carbon allocation patterns during internode sugar accumulation in different sorghum genotypes. Plant Biotech J. 2019; 17(2):472-487.
23. Eom JS, Chen LQ, Sosso D, Julius BT, Lin I, Qu XQ, Braun D, Frommer WB. SWEETs, transporters for intracellular and intercellular sugar translocation. Curr Opn Plant Biol. 2015; 25:53-62.
24. McCormick RF, Truong FK, Sreedasyam A, Jenkins J, Shu S, Sims D, Kennedy M, Amirebrahimi M, Weers BD, McKinley B, et al. The *Sorghum bicolor* reference genome: improved assembly, gene annotations, a transcriptome atlas, and signatures of genome organization. Plant J. 2018; 93(2):338-354.
