# Supplementary material for: Common metabolic networks contribute to carbon sink strength of sorghum internodes: implications for bioenergy improvement
Source: Biotechnol Biofuels. 2019 Nov 20;12:274. doi: 10.1186/s13068-019-1612-7 (PMC6868837; doi:10.1186/s13068-019-1612-7)
Supplement: Supplementary file 6 — Additional file 6. Annotation and nomenclature of SbSWEETs. [file 13068_2019_1612_MOESM6_ESM.docx]

**Additional file 6.** Sorghum SWEET genes and their homologs and orthologs in maize. The nomenclature of SbSWEETs from Mizuno *et al.* (2015) and Bihmidine *et al.* (2016) are provided. Maize homolog were obtained from MaizeGDB and the orthologs are based on Zhang *et al*. (2017).

| **geneID** | **Nomenclature in Mizuno *et al*.** | **Nomenclature in Bihimidine *et al*.** | **Clade** | **Maize homolog** | **Maize ortholog 1** | **Maize ortholog 2** |
| --- | --- | --- | --- | --- | --- | --- |
| Sobic.003G377700 | SbSWEET3-8 | SbSWEET1A | I | GRMZM2G039365 | none | none |
| Sobic.009G143500 | SbSWEET9-2 | SbSWEET1B | I | GRMZM2G153358 | none | none |
| Sobic.003G182800 | SbSWEET3-5 | SbSWEET2A | I | GRMZM2G324903 | none | none |
| Sobic.003G269300 | SbSWEET3-7 | SbSWEET2B | I | GRMZM6G056015 | none | none |
| Sobic.009G080900 | SbSWEET9-1 | SbSWEET3A | I | GRMZM2G179679 | none | none |
| Sobic.003G015200 | SbSWEET3-1 | SbSWEET3B | I | GRMZM2G060974 | none | none |
| Sobic.003G038700 | SbSWEET3-2 | SbSWEET_038700 | II | none | none | none |
| Sobic.003G038800 | SbSWEET3-3 | SbSWEET_038800 | II | none | none | none |
| Sobic.004G136600 | SbSWEET4-3 | SbSWEET4A | II | GRMZM2G000812 | none | none |
| Sobic.004G133500 | SbSWEET4-1 | SbSWEET4B | II | GRMZM2G144581 | none | none |
| Sobic.004G133600 | SbSWEET4-2 | SbSWEET4C | II | GRMZM2G137954 | none | none |
| Sobic.009G252000 | SbSWEET9-3 | SbSWEET5 | II | none | none | none |
| Sobic.003G213000 | SbSWEET3-6 | SbSWEET6 | II | none | GRMZM2G157675 | GRMZM2G416965 |
| Sobic.007G191200 | SbSWEET7-1 | SbSWEET11A | III | GRMZM2G368827 | none | none |
| Sobic.002G259300 | SbSWEET2-1 | SbSWEET11B | III | none | none | none |
| Sobic.001G373600 | SbSWEET1-1 | SbSWEET12 | III | none | GRMZM2G133322 | GRMZM2G099609 |
| Sobic.008G094000 | SbSWEET8-1 | SbSWEET13A | III | none | GRMZM2G173669 | GRMZM2G179349 |
| Sobic.008G094300 | SbSWEET8-2 | SbSWEET13B | III | none | GRMZM2G173669 | GRMZM2G179349 |
| Sobic.008G094400 | SbSWEET8-3 | SbSWEET13C | III | none | GRMZM2G021706 | GRMZM2G179349 |
| Sobic.005G123500 | SbSWEET5-1 | SbSWEET14 | III | none | GRMZM2G015976 | GRMZM2G094955 |
| Sobic.004G157100 | SbSWEET4-4 | SbSWEET15 | III | none | GRMZM5G872392 | GRMZM2G168365 |
| Sobic.001G377600 | SbSWEET1-2 | SbSWEET16 | IV | GRMZM2G107597 | none | none |
| Sobic.003G149000 | SbSWEET3-4 | SbSWEET17 | IV | none | GRMZM2G106462 | GRMZM2G111926 |
