# Supplementary material for: Common metabolic networks contribute to carbon sink strength of sorghum internodes: implications for bioenergy improvement
Source: Biotechnol Biofuels. 2019 Nov 20;12:274. doi: 10.1186/s13068-019-1612-7 (PMC6868837; doi:10.1186/s13068-019-1612-7)
Supplement: Supplementary file 7 — Additional file 7. Spatio-temporal expression patterns of sorghum SWEETs. [file 13068_2019_1612_MOESM7_ESM.docx]

**Additional file 7.** Spatio-temporal expression patterns of sorghum SWEETs. **(a)** Pfam domain search for Sobic.003G038700 and Sobic.003G038800 with SbSWEET11B (Sobic.002G259300) as a positive control, demonstrating Sobic.003G038700 but not Sobic.003G038800 has two intact MtN3 domains (criteria for identifying SWEET proteins according to Eom *et al.* 2015) (**b**) SbSWEETs expression patterns from the MOROKOSHI expression database. The expression levels of clade III SbSWEETs in stem are highlighted in red box; (**c**) SbSWEETs expression heatmap in BTx623 from the sorghum expression atlas (accessed from Phytozome) at various developmental stages and tissues. Heatmap is scaled according to Z-score normalized for each gene with RPKM expression values shown on the heatmap (McCormick *et al.* 2018). Additional file13. **b** and **c** clearly show spatio-temporal expression preference of the SbSWEETs.

**
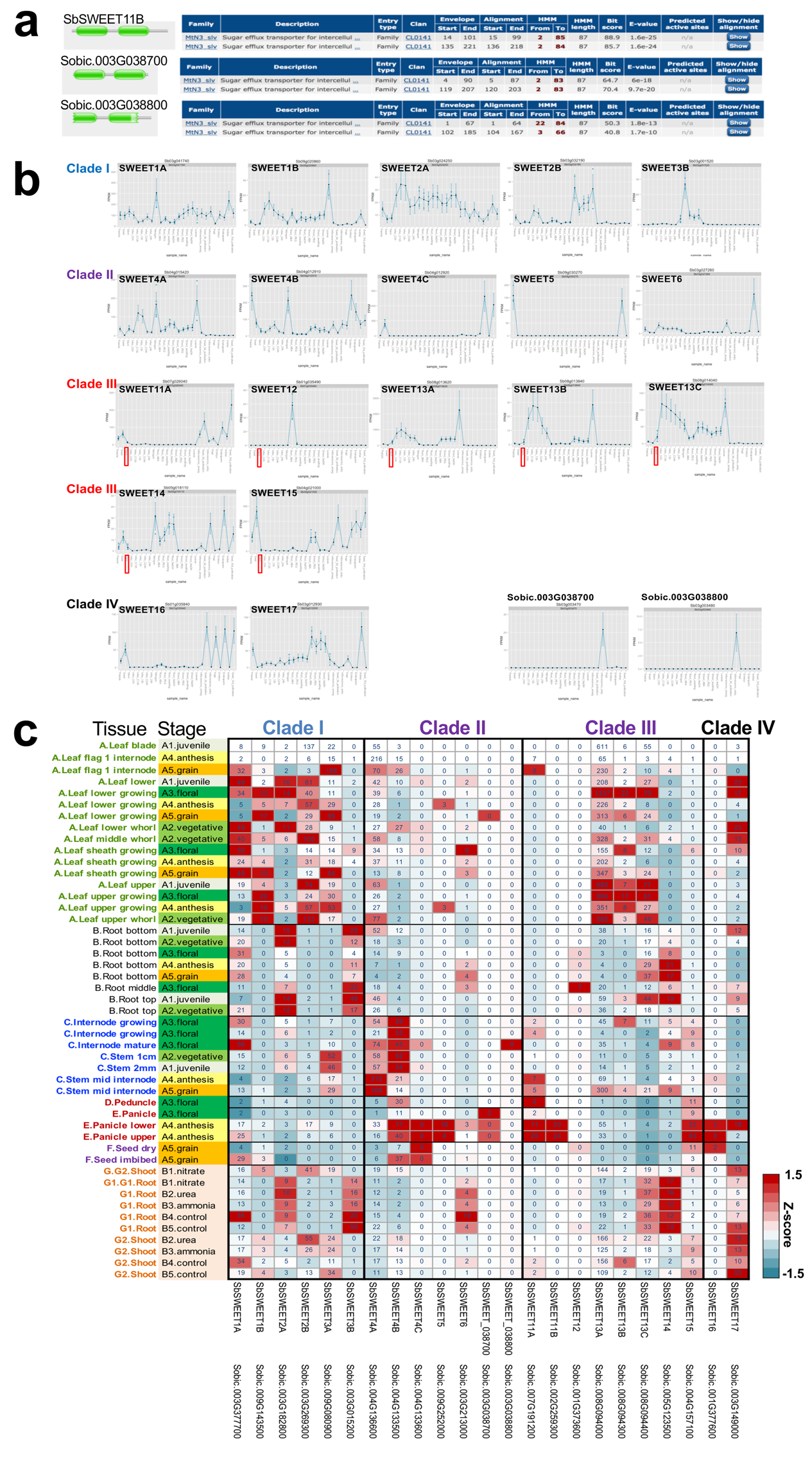
**
