# Supplementary material for: Common metabolic networks contribute to carbon sink strength of sorghum internodes: implications for bioenergy improvement
Source: Biotechnol Biofuels. 2019 Nov 20;12:274. doi: 10.1186/s13068-019-1612-7 (PMC6868837; doi:10.1186/s13068-019-1612-7)
Supplement: Supplementary file 8 — Additional file 8. Primers used for real-time quantitative PCR (qPCR). [file 13068_2019_1612_MOESM8_ESM.docx]

**Additional file 8.** Primers used for real-time quantitative PCR (qPCR).

| **GeneID** | **Gene name** | **Reference** | **Forward (5’-3’)** | **Reverse (5’-3’)** |
| --- | --- | --- | --- | --- |
| Sobic.004G062500 | 4CL | Scully *et al*. 2016 | CCGAAGGCTCTGAAGTCACCGAG | AGGATCTTGCCGGACGGGTTC |
| Sobic.004G071000 | CAD4 | Turco *et al*. 2017 | CTTTGTGCGTTCTTGCCGTGTTCT | CGCAGCACCAGAAACATTTGGGTA |
| Sobic.010G052200 | CCoAOMT | Scully *et al*. 2016 | AGATCACCGCCAAGCACCCA | GCGCCGATGAGCTTGATGAGC |
| Sobic.007G141200 | CCR | Saballos *et al*. 2012 | CTCAAGTACCTGGACGGCTC | GAACTTGTACGGCTGCTTCC |
| Sobic.007G047300 | COMT | Saballos *et al*. 2012 | GAGGACAAGGACGGCAAGTA | GTACTCGAACGCCGTCATC |
| Sobic.001G196300 | F5H | Agarwal *et al*. 2016 | CAACTGCCCCCTGTACTGAA | AGCCCTCTAAACTCCCCACA |
| Sobic.004G004800 | INV3 | Ghate *et al*. 2017 | CTCCCTCACCACGCACTTCT | GCGGAGCGAGAGCGTGG |
| Sobic.004G220300 | PAL | Scully *et al*. 2016 | TCTACGGCGTCACCACGGGG | ACCTCCGACGGCAGCGTGT |
| Sobic.008G193300 | SUT2 | Ghate *et al*. 2017 | TGGGCTGGTGGCTATTCTG | CCAGCCAACCGCAAAGAATT |
| Sobic.008G094000 | SWEET13A | Bihmidine *et al*. 2016 | CGCTCACTACTGCTAAGTATTAT | ACAGTAGTCTGGGATCGATTA |
| Sobic.004G099300 | TST2 | Bihmidine *et al*. 2016 | TTGGAGGTTGGAGGAGAC | CTTGGAAGGTCGAGCAATC |
| Sobic.001G100001 | AGP | This manuscript | TTCAGAGTCATCGGCAAAGAG | TGCAGTGTCGTCTATCTTCATTAG |
| Sobic.007G101500 | AGP | This manuscript | GATGCCTGGATTTGGGATACA | TCGTCACGCACAATAAGTAGAG |
| Sobic.002G116000 | GBSS1b | This manuscript | CCCTGAAACAGTATGACACCTC | GCACTTCCTCCCACTTCTTT |
| Sobic.010G047700 | SS | This manuscript | TCGTGGATGGGTTGGATTTAG | ATAGCTGATTGAGACCACAAGG |
| Sobic.007G204600 | ISA | This manuscript | CTATGCGGAAGTCCACAGTTAT | GACCAAATCAGCCAGTGTAAATC |
| Sobic.001G311100 | ubiquitin | Saballos *et al*. 2012 | GGTTCGGGAGGTGGCCTAGGT | AGCATGTACATTCCCAGCGGTAG |

**References for qPCR primers:**

Agarwal T, Grotewold E, Doseff AI, Gray J. MYB31/MYB42 Syntelogs Exhibit Divergent Regulation of Phenylpropanoid Genes in Maize, Sorghum and Rice. Sci Rep. 2016; 6:28502.

Bihmidine S, Julius BT, Dweikat I, Braun DM. Tonoplast Sugar Transporters SbTSTs. putatively control sucrose accumulation in sweet sorghum stems. Plant Signal Behav. 2016; 11:e1117721.

Ghate T, Deshpande S, Bhargava S. Accumulation of stem sugar and its remobilisation in response to drought stress in a sweet sorghum genotype and its near-isogenic lines carrying different stay-green loci. Plant Biol (Stuttg). 2017;19(3):396-405.

Saballos A, Sattler SE, Sanchez E, Foster TP, Xin Z, Kang C, Pedersen JF, *et al*. Brown midrib2 (Bmr2) encodes the major 4-coumarate: coenzyme A ligase involved in lignin biosynthesis in sorghum (Sorghum bicolor (L.) Moench). Plant J. 2012; 70(5):818-830.

Scully ED, Gries T, Sarath G, Palmer NA, Baird L, Serapiglia MJ, Dien BS, Boateng AA, Ge Z, Funnell-Harris DL, *et al.* Overexpression of SbMyb60 impacts phenylpropanoid biosynthesis and alters secondary cell wall composition in Sorghum bicolor. Plant J. 2016; 85(3):378-95.

Turco GM, Kajala K, Kunde-Ramamoorthy G, Ngan CY, Olson A, Deshphande S, Tolkunov D, Waring B, Stelpflug S, Klein P, *et al.* DNA methylation and gene expression regulation associated with vascularization in Sorghum bicolor. New Phytol. 2017; 214(3):1213-1229.
