# Supplementary material for: Common metabolic networks contribute to carbon sink strength of sorghum internodes: implications for bioenergy improvement
Source: Biotechnol Biofuels. 2019 Nov 20;12:274. doi: 10.1186/s13068-019-1612-7 (PMC6868837; doi:10.1186/s13068-019-1612-7)
Supplement: Supplementary file 9 — Additional file 9. Dynamics of internode Brix (a) and water concentration (b) over stem sugar accumulation. [file 13068_2019_1612_MOESM9_ESM.docx]

**Additional file 9.** Dynamics of internode Brix (**a**) and water concentration (**b**) over the time course of stem sugar accumulation in Rio and Della. The dynamics are shown in boxplots, with x-axis representing genotype and days after flowering (DAF). Within each internode, differences in Brix or water content were calculated by two-way ANOVA (genotype and time point as the two factors) followed by multiple comparison through the method of LSD and displayed by letter. Values with the same letter are not significantly different at *p*=0.05.

**
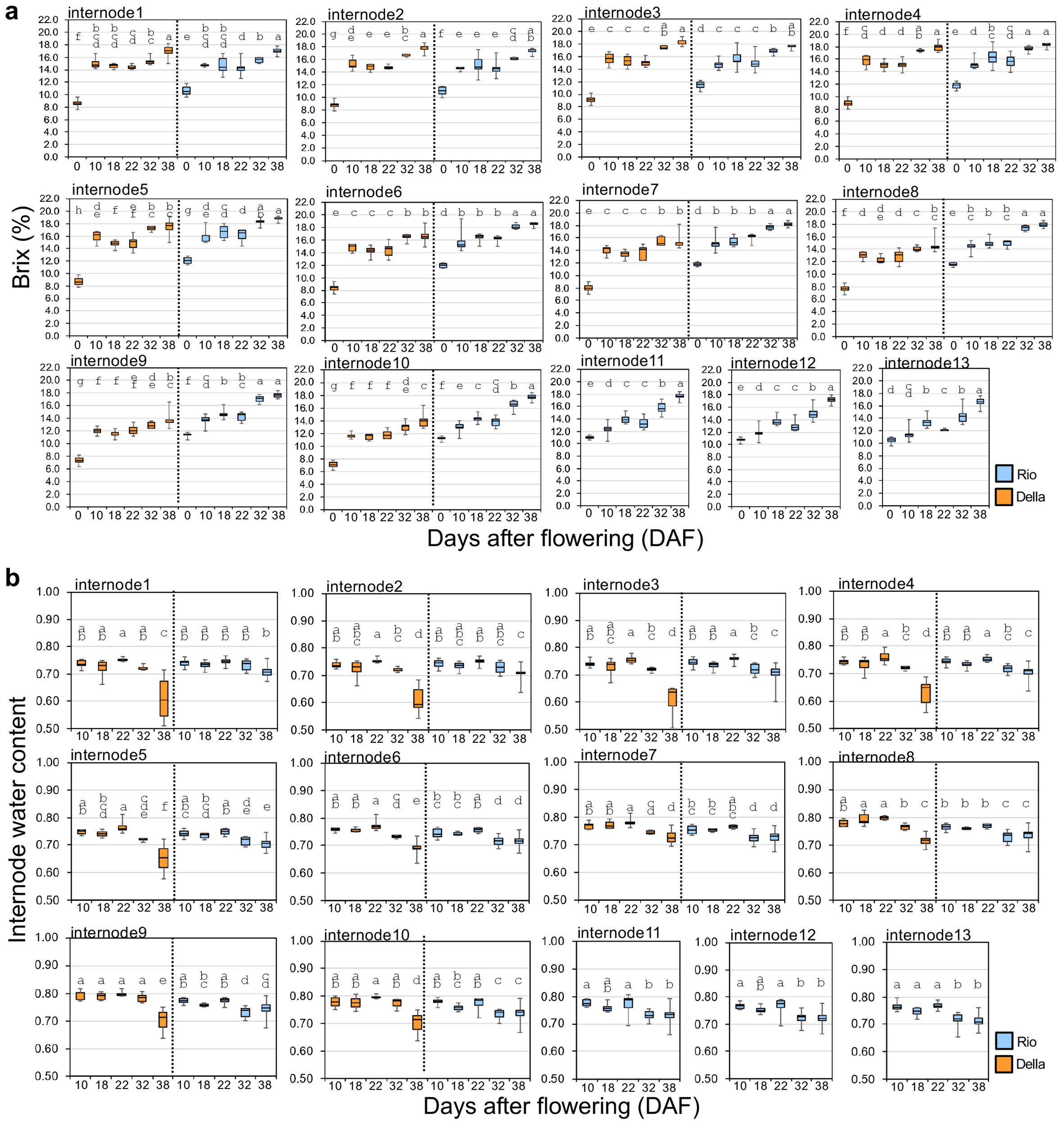
**
