# Supplementary material for: Common metabolic networks contribute to carbon sink strength of sorghum internodes: implications for bioenergy improvement
Source: Biotechnol Biofuels. 2019 Nov 20;12:274. doi: 10.1186/s13068-019-1612-7 (PMC6868837; doi:10.1186/s13068-019-1612-7)
Supplement: Supplementary file 10 — Additional file 10. Distribution of the expression levels for all the RNA-seq data sets used before (a) and after (b) batch-effect removal. [file 13068_2019_1612_MOESM10_ESM.docx]

**Additional file 10.** Distribution of the expression levels for all the RNA-seq data sets used before (a) and after (b) batch-effect removal.

**
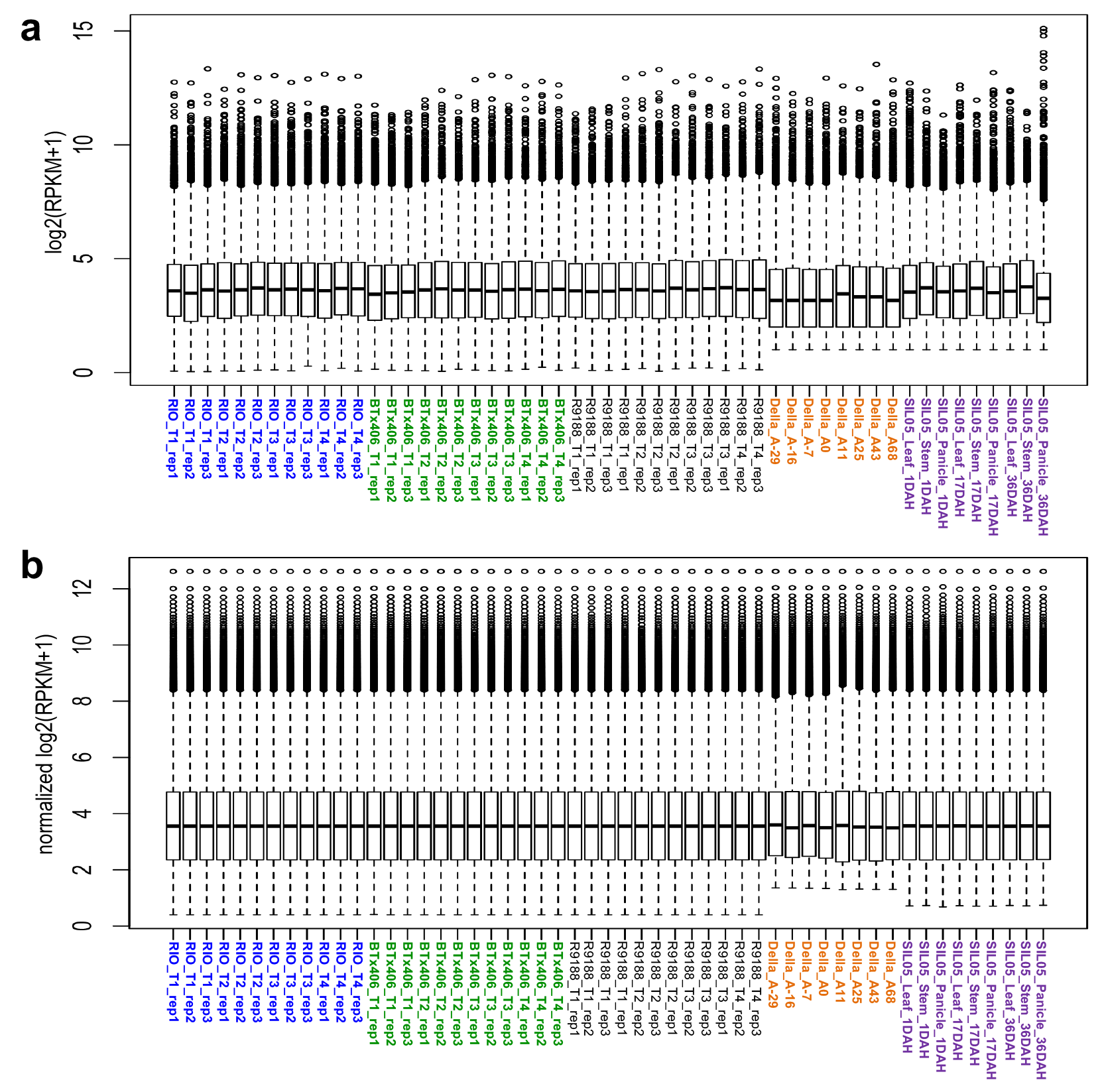
**
