# Supplementary figures and images for: Common metabolic networks contribute to carbon sink strength of sorghum internodes: implications for bioenergy improvement
Source: Biotechnol Biofuels. 2019 Nov 20;12:274. doi: 10.1186/s13068-019-1612-7 (PMC6868837; doi:10.1186/s13068-019-1612-7)

**Additional file 11.** Hierarchical clustering (HC) of the RNA-seq data sets used.

**
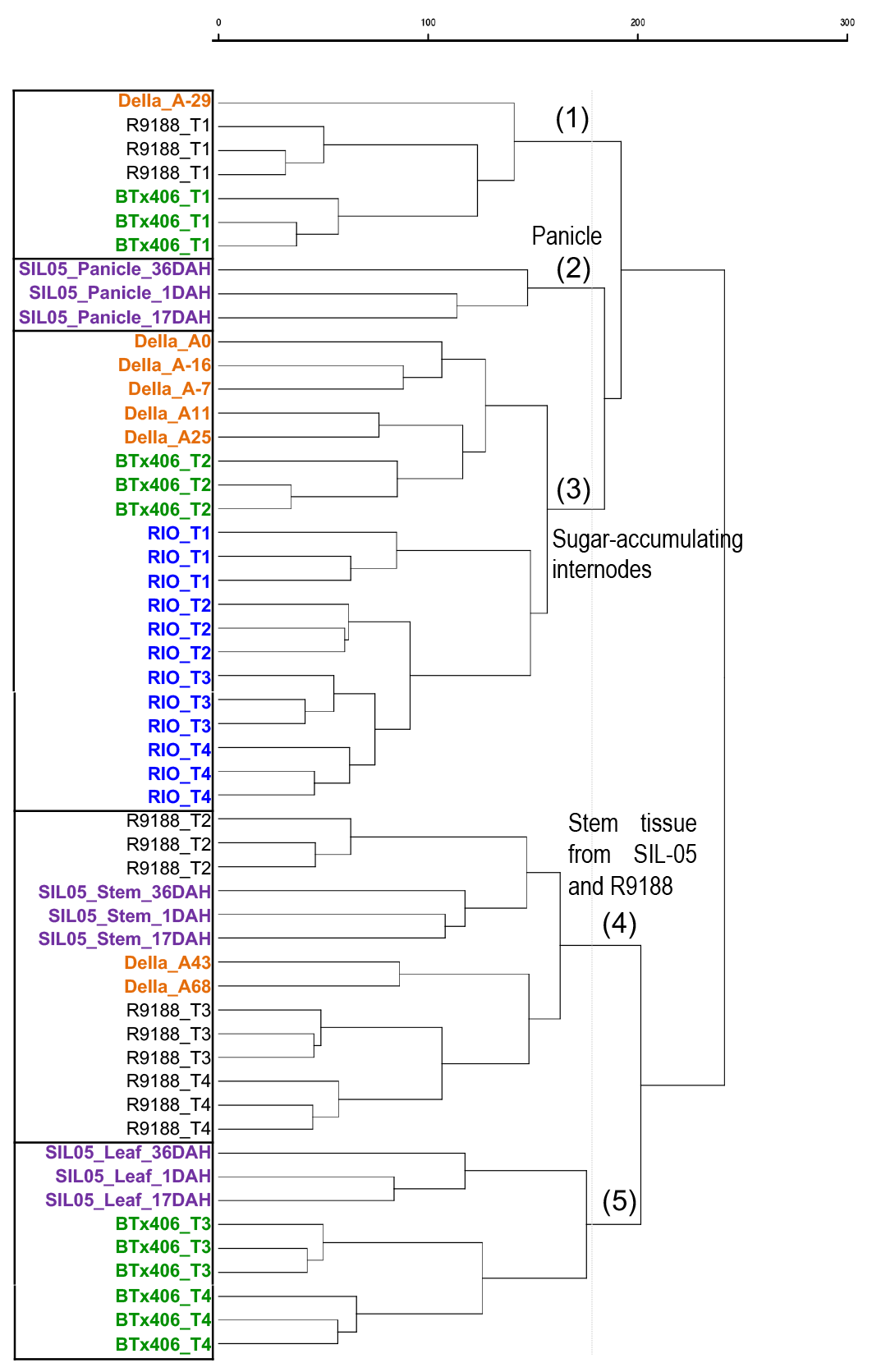
**

Supplement: Supplementary file 11 — Additional file 11. Hierarchical clustering (HC) of the RNA-seq data sets used. [file 13068_2019_1612_MOESM11_ESM.docx]
