# Supplementary material for: Common metabolic networks contribute to carbon sink strength of sorghum internodes: implications for bioenergy improvement
Source: Biotechnol Biofuels. 2019 Nov 20;12:274. doi: 10.1186/s13068-019-1612-7 (PMC6868837; doi:10.1186/s13068-019-1612-7)

**Additional file 13.** The dynamics of tyrosine and SAM (a) and the expression levels of genes involved in SAM metabolism (b).

SAM metabolism pathway is shown in **(c)**. Tyrosine and SAM contents were determined by non-target metabolomics in Rio, BTx406 and R9188 (n=6). The statistical differences in tyrosine and SAM between time points and genotypes were determined by ANOVA and Tukey’s test (*p*<0.05) and displayed by letter. The Rio vs BTx406 and Rio vs R9188 difference in both metabolites were also tested by Welch two-sample *t*-test (*, *p*<0.05; **, *p*<0.01; ***, *p*<0.005). Gene expression dynamics are shown in barplots, with x-axis representing the time points of each genotype, y-axis representing RPKM values, and error bar representing SEM. Within each genotype, gene differential expression (DE) compared between a time point and the corresponding pre-anthesis stage or the anthesis stage is labeled by black and red asterisks, respectively (DE criteria: *q* value<0.05 and log_2_FC >1; *, *q*<0.05; **, *q*<0.01; ***, *q*<0.005). For the RNA-seq results of BTx406, R9188 and Rio, expression differences between genotypes and time points were calculated by two-way ANOVA followed by multiple comparison and displayed by letter. Expression levels with the same letter are not significantly different at *p*=0.05. Gene IDs highlighted in red are those differentially expressed or with distinct expression trends between sweet and non-sweet genotypes.

SAMS: SAM synthase; SAHH: S-adenosyl-L-homocysteine hydrolase; HMT: homocysteine S-methyltransferase.


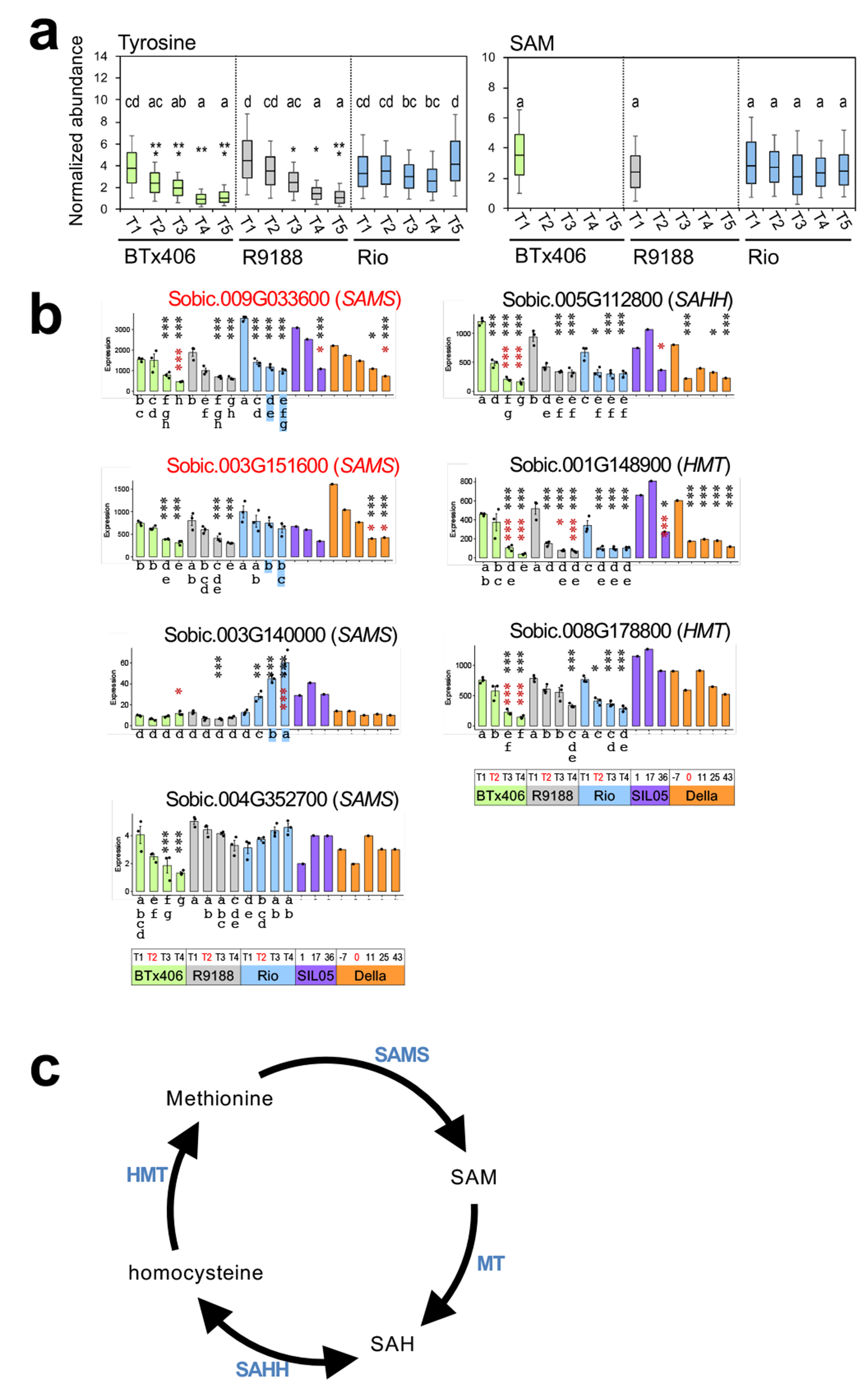

Supplement: Supplementary file 13 — Additional file 13. The dynamics of tyrosine and SAM and the expression levels of genes involved in SAM metabolism. [file 13068_2019_1612_MOESM13_ESM.docx]
