# Supplementary material for: Common metabolic networks contribute to carbon sink strength of sorghum internodes: implications for bioenergy improvement
Source: Biotechnol Biofuels. 2019 Nov 20;12:274. doi: 10.1186/s13068-019-1612-7 (PMC6868837; doi:10.1186/s13068-019-1612-7)
Supplement: Supplementary file 14 — Additional file 14. Annotation of sorghum invertases (INVs) and their homologs and orthologs in maize. [file 13068_2019_1612_MOESM14_ESM.docx]

**Additional file 14.** Annotation of sorghum invertases (INVs) and their homologs and orthologs in maize. na= not applicable; The numbering of each geneID within the INV subfamilies is in accordance with Wang et al. 2017.

| **geneID in Sorghum (v3.1)** | **geneID in Sorghum**  **(v2.1)** | **Homolog in Maize** |  | **gene name** | **INV type** | **Sorghum INV nomenclature** | **Ortholog in Maize** |  | **Other comments** |
| --- | --- | --- | --- | --- | --- | --- | --- | --- | --- |
|  |  | **geneID (v4) in Maize** | **geneID (v3) in Maize** |  |  |  | **Zm1_ortholog1** | **Zm2_ortholog2** |  |
| Sobic.004G255600 | Sobic.004G255600 | Zm00001d051666 | GRMZM2G136139 | invertase | INVAN | SbINVAN2 | No Gene | GRMZM2G136139 | na |
| Sobic.003G153800 | Sobic.003G153800 | Zm00001d040735 | GRMZM2G084694 | invertase | INVAN | SbINVAN6 | GRMZM2G084694 | GRMZM2G022782 | na |
| Sobic.004G172700 | Sobic.004G172700 | Zm00001d016768 | GRMZM2G118737 | invertase | INVAN | SbINVAN1 | GRMZM2G118737 | GRMZM2G170842 | na |
| Sobic.005G058800 | Sobic.005G058800 | Zm00001d053017 | GRMZM2G007277 | invertase | INVAN | SbINVAN3 | GRMZM2G007277 | No Gene | na |
| Sobic.004G024500 | Sobic.004G024500 | Zm00001d015094 | GRMZM2G477236 | invertase | INVAN | SbINVAN4 | GRMZM2G477236 | No Gene | na |
| Sobic.001G391600 | Sobic.001G391600 | Zm00001d028926 | GRMZM2G084940 | invertase | INVAN | SbINVAN7 | GRMZM2G084940 | No Gene | na |
| Sobic.004G163800 | Sobic.004G163800 | Zm00001d050645 | GRMZM2G040843 | invertase | INVAN | SbINVAN5 | No Gene | No Gene | na |
| Sobic.004G166700 | Sobic.004G166700 | Zm00001d016708 | GRMZM2G139300 | invertase | INVCW | SbINVCW4 | GRMZM2G139300 | No Gene | na |
| Sobic.001G099700 | Sobic.001G099700 | Zm00001d025355 | GRMZM2G123633 | invertase | INVCW | SbINVCW1 | No Gene | No Gene | na |
| Sobic.006G070298 | Sobic.K041000 | Zm00001d003776 | GRMZM2G119689 | invertase | INVCW | SbINVCW5 | GRMZM2G119689 | GRMZM2G095725 | expressed in panicle and root |
| Sobic.006G070830 | Sobic.K041200 | Zm00001d003776 | GRMZM2G119689 | invertase | INVCW | SbINVCW6 | GRMZM2G119689 | GRMZM2G095725 | expressed in panicle |
| Sobic.006G070032 | Sobic.K040900 | Zm00001d025355 | GRMZM2G123633 | invertase | INVCW | SbINVCW2 | GRMZM2G119689 | GRMZM2G123633 | expressec in panicle |
| Sobic.006G255600 | Sobic.006G255600 | Zm00001d001941 | GRMZM2G119941 | invertase | INVCW | SbINVCW10 | GRMZM2G119941 | No Gene | na |
| Sobic.006G070564 | Sobic.K041100 | Zm00001d025354 | GRMZM2G095725 | invertase | INVCW | SbINVCW3 | GRMZM2G119689 | GRMZM2G095725 | expressed in panicel and root |
| Sobic.006G255400 | Sobic.006G255400 | Zm00001d001944 | GRMZM2G018692 | invertase | INVCW | SbINVCW9 | GRMZM2G018692 | No Gene | na |
| Sobic.006G255500 | Sobic.006G255500 | Zm00001d001943 | GRMZM2G018716 | invertase | INVCW | SbINVCW8 | GRMZM2G018716 | No Gene | na |
| Sobic.003G440900 | Sobic.003G440900 | Zm00001d041991 | GRMZM2G174249 | invertase | INVCW | SbINVCW7 | GRMZM2G174249 | No Gene | na |
| Sobic.006G160700 | Sobic.006G160700 | Zm00001d002830 | GRMZM2G394450 | invertase | INVVR | SbINVVR2 | GRMZM2G394450 | No Gene | na |
| Sobic.004G004800 | Sobic.004G004800 | Zm00001d014947 | GRMZM2G089836 | invertase | INVVR | SbINVVR1 | GRMZM2G089836 | GRMZM2G463871 | na |
