# Supplementary material for: Common metabolic networks contribute to carbon sink strength of sorghum internodes: implications for bioenergy improvement
Source: Biotechnol Biofuels. 2019 Nov 20;12:274. doi: 10.1186/s13068-019-1612-7 (PMC6868837; doi:10.1186/s13068-019-1612-7)

**Additional file 16.** The phylogenetic tree of SWEET proteins from rice, maize and sorghum.

The phylogenetic analysis validated the four clades defined by a previous study [53], which is well correlated to the substrate preference. Black dots and crosses highlight the expressed and non-expressed clade III *SbSWEET*s, respectively, in the RNA-seq samples used here.


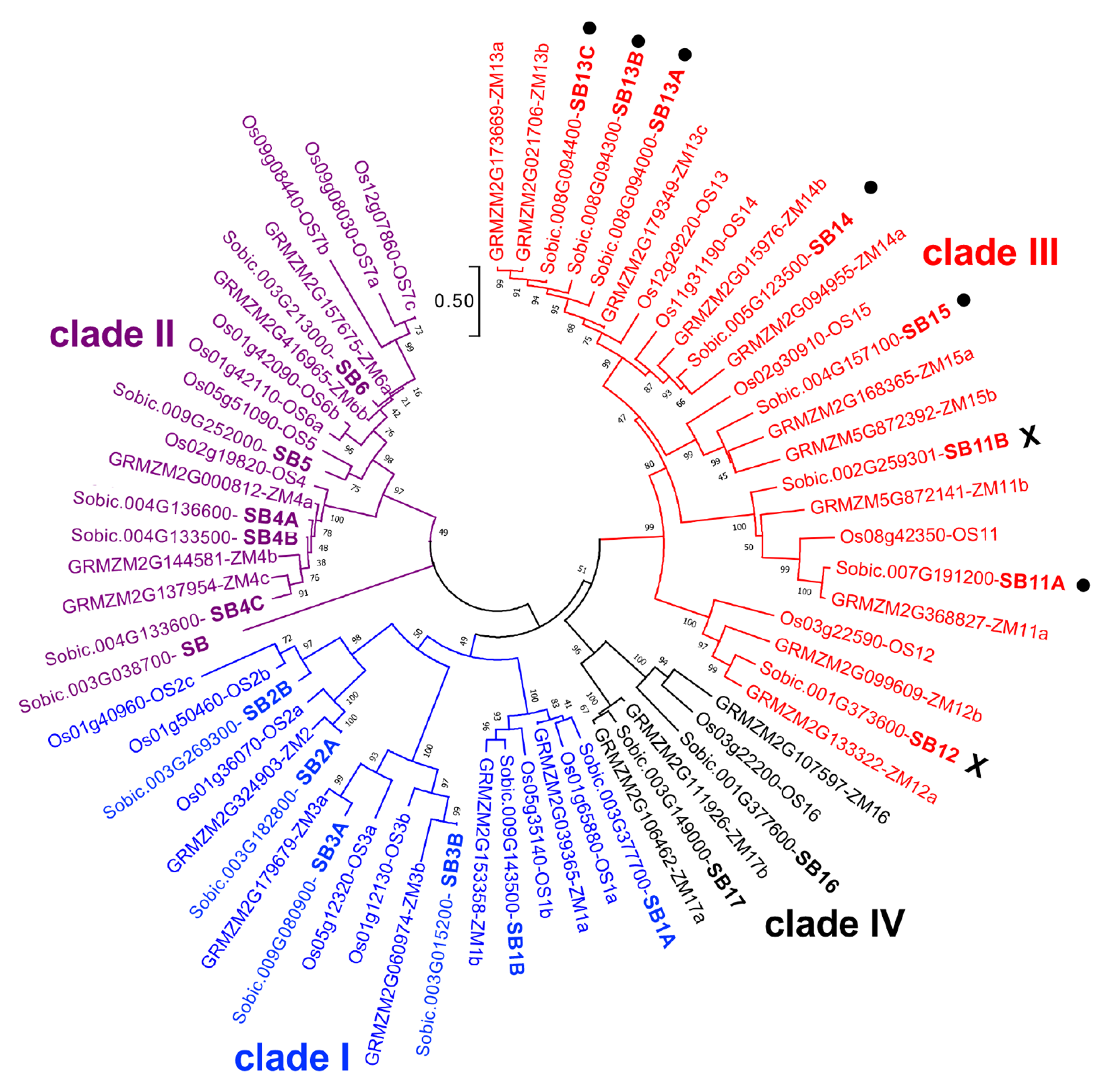

Supplement: Supplementary file 16 — Additional file 16. The phylogenetic tree of SWEET proteins from rice, maize and sorghum. [file 13068_2019_1612_MOESM16_ESM.docx]
