# Supplementary material for: Common metabolic networks contribute to carbon sink strength of sorghum internodes: implications for bioenergy improvement
Source: Biotechnol Biofuels. 2019 Nov 20;12:274. doi: 10.1186/s13068-019-1612-7 (PMC6868837; doi:10.1186/s13068-019-1612-7)

**Additional file 17.** Validation of RNA-seq results by qRT-PCR.

The expression levels of the sixteen genes were determined by 2^−(ΔΔCt)^ method and normalized to the Rio T1 stage. The similarity between qPCR and RNA-seq expression patterns was calculated using *Pearson* correlation with correlation coefficient (*r*) and *P* values given in the figure. RNA-seq expression values were normalized to that of Ubiquitin (Sobic. 001G311100) at Rio T1 stage. The RNA-seq results are labeled using dotted boxes. For the qPCR results of BTx406, R9188 and Rio, expression differences between genotypes and time points were calculated by two-way ANOVA followed by multiple comparison and displayed by letter. Expression levels with the same letter are not significantly different at *p*=0.05.


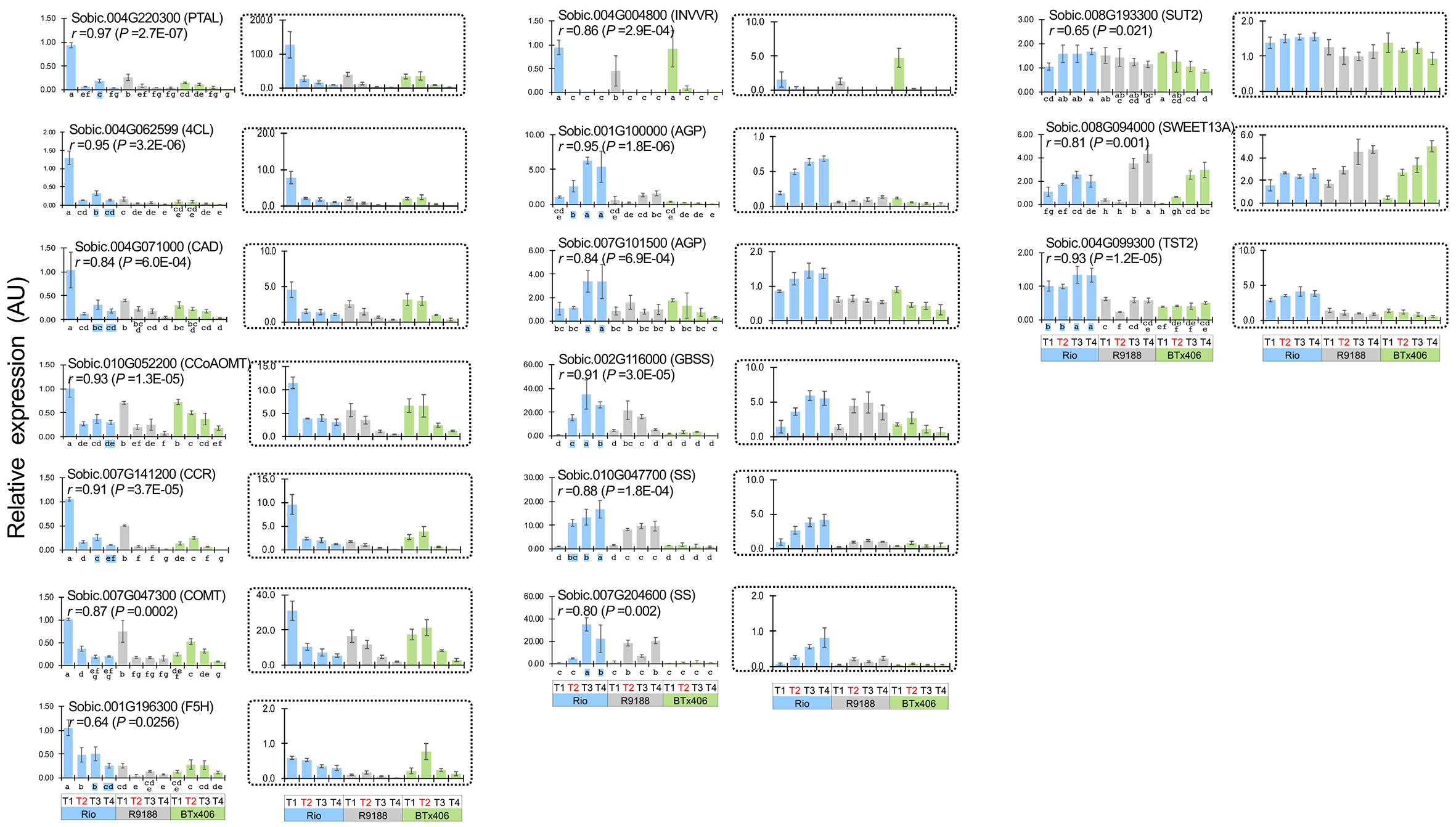

Supplement: Supplementary file 17 — Additional file 17. Validation of RNA-seq results by qPCR. [file 13068_2019_1612_MOESM17_ESM.docx]
