# Supplementary material for: Common metabolic networks contribute to carbon sink strength of sorghum internodes: implications for bioenergy improvement
Source: Biotechnol Biofuels. 2019 Nov 20;12:274. doi: 10.1186/s13068-019-1612-7 (PMC6868837; doi:10.1186/s13068-019-1612-7)

**Additional file 18.** A proposed model for carbon partitioning and sink strength in sorghum internodes.

The graphic model depicts the carbon allocation in sorghum internodes that are likely composed of four major routes: sucrose metabolism, sucrose transported and stored in vacuoles, starch metabolism and cell wall-related metabolism. The four grey arrows represent the four carbon utilization routes, with arrow width qualitatively corresponds to the carbon fractions partitioned in each route. Detailed interpretation of carbon allocation is given in the discussion. The model also illustrates the difference in carbon allocation between sweet and non-sweet genotypes, with the candidate genes associated with these differential metabolic pathways colored based on their regulation during sugar accumulation.


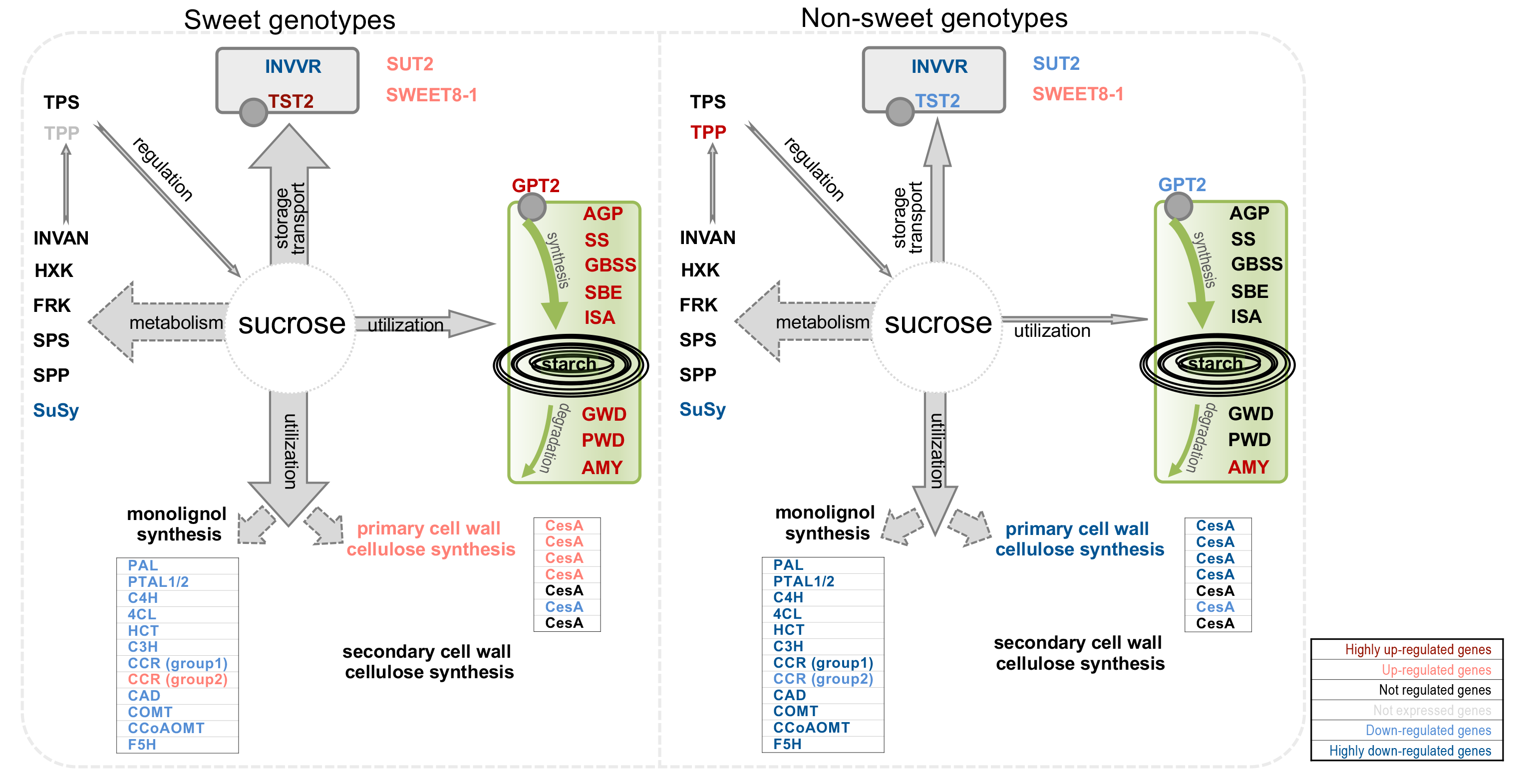

Supplement: Supplementary file 18 — Additional file 18. A proposed model for carbon partitioning and sink strength in sorghum internodes. [file 13068_2019_1612_MOESM18_ESM.docx]
